# Isolation of Ciprofloxacin and Ceftazidime-Resistant Enterobacterales From Vegetables and River Water Is Strongly Associated With the Season and the Sample Type

**DOI:** 10.3389/fmicb.2021.604567

**Published:** 2021-09-14

**Authors:** Constanza Díaz-Gavidia, Carla Barría, Lina Rivas, Patricia García, Francisca P. Alvarez, Gerardo González-Rocha, Andrés Opazo-Capurro, Rafael Araos, José M. Munita, Sandra Cortes, Jorge Olivares-Pacheco, Aiko D. Adell, Andrea I. Moreno-Switt

**Affiliations:** ^1^Escuela de Medicina Veterinaria, Facultad de Ciencias de la Vida, Universidad Andres Bello, Santiago, Chile; ^2^Millennium Initiative for Collaborative Research on Bacterial Resistance (MICROB-R), Santiago, Chile; ^3^Genomics and Resistant Microbes Group, Facultad de Medicina Clínica Alemana, Universidad del Desarrollo, Santiago, Chile; ^4^Escuela de Medicina, Facultad de Medicina, Pontificia Universidad Católica de Chile, Santiago, Chile; ^5^Laboratorio de Investigación en Agentes Antibacterianos (LIAA), Departamento de Microbiología, Facultad de Ciencias Biológicas, Universidad de Concepción, Concepción, Chile; ^6^Advance Center for Chronic Diseases (ACCDiS), Escuela de Medicina, Pontificia Universidad Católica de Chile, Santiago, Chile; ^7^Centro de Desarrollo Urbano Sustentable, Pontificia Universidad Católica de Chile, Santiago, Chile; ^8^Grupo de Resistencia Antimicrobiana en Bacterias Patógenas y Ambientales (GRABPA), Instituto de Biología, Pontificia Universidad Católica de Valparaíso, Valparaíso, Chile; ^9^Escuela de Medicina Veterinaria, Facultad de Agronomía e Ingeniería Forestal, Facultad de Ciencias Biológicas y Facultad de Medicina, Pontificia Universidad Católica de Chile, Santiago, Chile

**Keywords:** multidrug resistance, vegetable, river water, Chile, environmental risk factors, Enterobacterales

## Abstract

The dissemination of antibiotic-resistant bacteria (ARB) from water used for crop irrigation to vegetables is poorly studied. During a year, five farmer markets in a city in Central Chile were visited, and 478 vegetable samples (parsleys, corianders, celeries, lettuces, chards, and beets) were collected. Simultaneously, 32 water samples were collected from two rivers which are used to irrigate the vegetables produced in the area. Resistant Enterobacterales were isolated and identified. Colistin resistance gene *mcr-1* and extended spectrum β-lactamases (ESBL) were molecularly detected. The association of environmental factors was evaluated, with the outcomes being the presence of Enterobacterales resistant to four antibiotic families and the presence of multidrug resistance (MDR) phenotypes. Parsley, coriander, and celery showed the highest prevalence of resistant Enterobacterales (41.9% for ciprofloxacin and 18.5% for ceftazidime). A total of 155 isolates were obtained, including *Escherichia coli* (*n*=109), *Citrobacter* sp. (*n*=20), *Enterobacter cloacae* complex (*n*=8), *Klebsiella pneumoniae* (*n*=8), and *Klebsiella aerogenes* (*n*=1). Resistance to ampicillin (63.2%) and ciprofloxacin (74.2%) was most frequently found; 34.5% of the isolates showed resistance to third-generation cephalosporins, and the MDR phenotype represented 51.6% of the isolates. In two *E. coli* isolates (1.29%), the gene *mcr-1* was found and ESBL genes were found in 23/62 isolates (37%), with *bla*_CTX-M_ being the most frequently found in 20 isolates (32%). Resistant Enterobacterales isolated during the rainy season were less likely to be MDR as compared to the dry season. Understanding environmental associations represent the first step toward an improved understanding of the public health impact of ARB in vegetables and water.

## Introduction

Antimicrobial resistance (AMR) is one of the most pressing global public health concerns and has been prioritized by the WHO, World Health Organization (2019). The AMR crisis is directly related to the overuse and misuse of antibiotics in multiple activities, including human health, animal production, and agriculture (Manage and Liyanage, 2019). Factors like environmental pollution and livestock production practices likely accelerate the dissemination of antibiotic-resistant bacteria (ARB) and antimicrobial resistance genes (ARGs) in the environment (e.g., water and soil), facilitating food contamination (Hernando-Amado et al., 2019). Understanding the specific role of each of these factors in the dispersion of ARB and/or ARGs is critical to tackle the fast increase of AMR (Hernando-Amado et al., 2019).

Extended spectrum β-lactamase (ESBL)-producing Enterobacterales, such as *Escherichia coli* and *Klebsiella pneumoniae*, are among the top AMR threats (Rajendran et al., 2019). Indeed, the WHO ranked ESBL-producing and carbapenem-resistant Enterobacterales within the critically important pathogens, against which novel strategies are urgently needed (WHO, World Health Organization, 2019). Infections due to ESBL-producing Enterobacterales result in 197,400 hospitalizations and 9,100 deaths per year only in the United States (CDC, Centers for Disease Control and Prevention, 2019). In addition, these pathogens have also been reported in numerous animals, including livestock (poultry and cattle) and companion animals (cats and dogs) (Saliu et al., 2017; Melo et al., 2018; Dantas Palmeira and Ferreira, 2020). In the same way, ESBL-producing Enterobacterales have been found in food (Ye et al., 2018) and water sources (Tanner et al., 2019), highlighting their dissemination into the environment.

In terms of food safety, most of the interest in AMR has focused on recognized foodborne pathogens (e.g., *Salmonella* spp. and *Campylobacter* spp.). Not only have these shown a considerable rise in the overall levels of AMR, but also there are increasing reports of emerging resistance to third-generation cephalosporins (3GC) and other critical antibiotics (e.g., colistin and carbapenems) (Lima et al., 2019). Less attention has been given to the presence of clinically relevant ARB in food, whereas current evidence suggests that their presence could be widespread in food products (Hudson et al., 2017). For instance, studies conducted on leafy greens and vegetables have described the presence of multidrug resistance (MDR) Enterobacterales (Zekar et al., 2017; Hölzel et al., 2018; Sapkota et al., 2019; Oh et al., 2020). Similarly, Saksena et al. (2020) described the presence of resistant Enterobacterales, including *Klebsiella* spp., *Escherichia coli*, *Citrobacter* spp., and *Enterobacter* spp., in a wide range of fruits and vegetables (e.g., apples, tomatoes, and cucumbers, among others) (Saksena et al., 2020). Importantly, previous studies have shown that most of the ARB recovered from vegetables presented resistance to 3GC. In addition, another study reported the presence of fluoroquinolone-resistant *E. coli* in cabbage, lettuce, and spinach in South Africa (Jongman and Korsten, 2016).

Contaminated water used to irrigate crops is one of the main contributors to vegetable contamination with ARB and ARGs (Iwu and Okoh, 2019). This phenomenon has been attributed not only to surface water but also to water wells and groundwater, which have been shown to be capable of transmitting ARB to vegetables and animals (Wu et al., 2016; Guo et al., 2019). Antimicrobial-resistant Enterobacterales have been reported in surface water, with MDR *E. coli* being the most common bacteria detected in a previous study conducted on the Caribbean island of Guadeloupe, where areas with a low population density showed lower numbers of resistant *E. coli* in comparison with highly populated urban areas (Guyomard-Rabenirina et al., 2017). While several factors could influence water contamination with ARB, including climate, land usage, and urbanization (Yuan et al., 2019; Weller et al., 2020), in general, the main sources of contamination are feces from humans and animals, including run-off from farms (feed-lots or dairy), manure used as fertilizer, and waste from water treatment plants (Liu et al., 2013; Luna-Guevara et al., 2019). Importantly, in numerous developing countries, rural areas lack drinking water and proper sewage disposal. Consequently, human waste material accumulates in surface water (rivers) (Ferronato and Torretta, 2019). In these circumstances, the use of surface water to irrigate crops is particularly relevant as a potential source of ARB dissemination, particularly in vegetables that are consumed raw or without proper cooking.

While previous studies have highlighted the potential risk to humans of water and vegetables contaminated with ARB, little is currently known about the environmental factors associated with the presence of ARB in water and vegetable sources. Hence, this study aims to determine the presence of clinically relevant antibiotic-resistant Enterobacterales in vegetables and surface water in an agricultural town in central Chile, and their association with environmental factors like season: dry and rainy; source: vegetable type and river water; ambient temperature; produce state (fresh, partly rotten, or completely rotten); presence of insects in farmer market sampled; produce stored at ground level; rain event 5days before sample collection; pest control present in farmer market; seller wearing gloves when manipulating vegetables; vegetables stored separately by type; and pets present in farmer market.

## Materials and Methods

### Study Location

**Figure 1:**
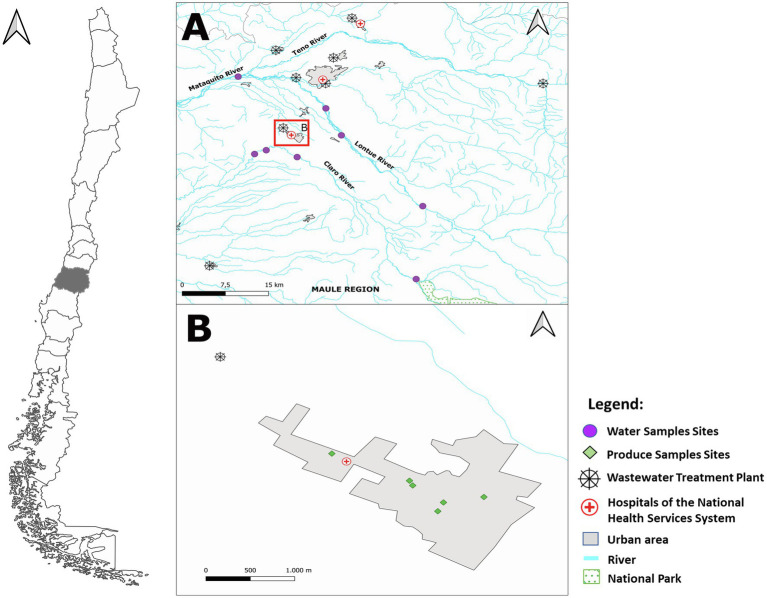
Map of the location of the study. **(A)** Sampling sites at the two parallel rivers (Claro River and Lontue Basins), **(B)** sites of the city of Molina in which the five farmer markets were sampled. In the figure, there are six sites for produce sampling because one of the markets moved to another location during the year.

This study was carried out in Molina, a city of approximately 45,000 inhabitants in central Chile (Figure 1). Molina is located in a region that comprises 17.2% of the national surface for agricultural and livestock production, from which the main activities are the production of crops, such as cereals, fruit trees, and vineyards (ODEPA, Oficina de Estudios y Políticas Agrarias, 2018). There are 761,981.2 hectares of land used for crop activities (ODEPA, Oficina de Estudios y Políticas Agrarias, 2018), mainly apples, cherries, and kiwis (ODEPA, Oficina de Estudios y Políticas Agrarias, 2018). Also, there are numerous small and medium-sized farms with backyard flocks and other animals, and small- and medium-scale production of vegetables (e.g., lettuce and spinach). Irrigation mainly derives from surface water obtained from two rivers (Lontue and Claro rivers) running parallel to the northern and southern borders of Molina (Figure 1A). The most frequently used irrigation system by small and medium producers is primary and secondary open furrows, using untreated river water distributed by different canal systems. The study area contains one main wastewater treatment plant (WWTP) that discharges the treated effluent into the Carretones Creek which discharges into the river beyond the sampling sites and area evaluated; therefore, this creek was not sampled. The region has a Mediterranean climate, with four seasons, characterized by rainy winters and falls, and dry springs and summers.

### Vegetable Samplings

To determine the prevalence of ARB in vegetables harvested and commercialized in the city of Molina, we selected the following vegetables: parsleys, lettuces, beets, chards, celeries, and corianders. This selection fulfilled the following criteria: (i) year-round cultivation in the region, (ii) raw consumption, and (iii) cultivated at ground level. These vegetables were locally grown by small, not industrialized farmers, and had a variety of distribution channels, from collection in larger markets and resale to direct sale in small farmer market. In this location, traceability of sold vegetables is either minimal or absent. Five farmer market selling locally harvested vegetables were selected (Figure 1B and Supplementary Figure S1). Four sampling efforts were performed between May 2019 and January 2020. Samplings were collected in May (fall), July (winter), both represented the rainy season, November (spring) and January (summer), both represented the dry season. A total of 478 vegetable samples were collected, including parsleys (*n*=103), lettuces (*n*=132), beets (*n*=114), chards (*n*=108), celeries (*n*=6), and corianders (*n*=15). In the absence of a given vegetable, these were replaced by a similar one (e.g., parsley was replaced by coriander). Vegetables were collected on the same market in each sampling; samples were maintained in sterile zip-locked plastic bags and transported aseptically to the Laboratory at Universidad Andres Bello. They were kept at 4°C and processed within 48h.

The following environmental factors were registered by samplers during each sampling effort: (i) ambient temperature (°C), (ii) produce state (fresh, partly rotten, or completely rotten), (iii) presence of insects in farmer market sampled (yes/no), (iv) produce stored at ground level (yes/no), (v) rain event 5days before sample collection (yes/no), (vi) pest control presented in farmer market, (vii) seller wearing gloves when manipulating vegetables, (viii) vegetables stored separately by type (yes/no), and (ix) pets present in farmer market (yes/no).

### Water Sampling and Processing

Water sampling was conducted in the course of the Claro and Lontue rivers, both of which originate in the Andes Mountains. The Lontue basin is composed of three river branches (Lontue, Mataquito, and Teno) and the Claro river flows, but flow into the Maule river. All of them finally flow into the Pacific Ocean (Figure 1A).

The Lontue and Claro rivers fulfilled the following characteristics: (i) run close to the location where vegetables were collected and produced; (ii) used as irrigation for agricultural purposes; (iii) have perennial flow; and (iv) provide secure access for researchers on foot and/or motor vehicle to collect samples. We sampled these rivers as their water is used to irrigate the area where the vegetables are produced. Sampling points corresponded to areas where each river crossed communes with population densities between 30.28 and 111.88habs/km^2^ (INE, Instituto Nacional de Estadísticas, 2017), including small populated areas with a few households and a middle-sized city. All sampling sites were georeferenced (Figure 1A).

A total of 32 water samples were collected from four sampling sites per river every 3months between May 2019 and January 2020, likewise vegetable samples, two visits represented the dry season, and two visits represented the rainy season. The first sampling site was located in the highest level at which water samples could be collected in the Andes Mountains to determine the original state of the river. The second site was located before the city where vegetables were collected for this study, while the third and fourth sampling sites were located after the city. As the farms of origin of the vegetables collected in the farmers market are scattered along the entire area, we selected different sites along the river’s course to evaluate the water quality used for irrigation. Experimentally, 10 liters of river water were collected using the Modified Moore Swab water filtration method (Sbodio et al., 2013). Briefly, this filtration system incorporates a filter gauze into a cassette to retain bacteria from the water sample as it is passed through using a portable peristaltic pump. All cassettes were kept in individual sterile zip-locked plastic bags, stored at 4°C, and transported aseptically to the Laboratory at Universidad Andres Bello, in Santiago, for processing.

The physical parameters of the river water—pH, water temperature (°C), conductivity (μs), salinity (ppm), and total dissolved solvents (ppm)—were measured and recorded *in situ* at each site using the Yalitech AM 006 Waterproof Multiparameter Meter Combo 6. Also, environmental factors from each visit were registered as: weather conditions (sunny, rainy, partly cloudy, and cloudy), rain event 5days prior to sample collection (yes/no), ambient temperature (continuous variable °C), presence of visible feces (yes/no), visible presence of domestic animals (yes/no), presence of garbage in sampling site (yes/no), observation of crops nearby sampling site (yes/no), and presence of aquatic plants in sampling sites (yes/no). These variables were further analyzed as described below.

### Bacterial Isolation and Identification

A total of 25*g* of each vegetable were obtained with a sterile scalpel and placed in bags containing 225ml of buffered peptone water (BD, Franklin Lakes, NJ, United States). Samples were homogenized in a stomacher (IUL Instruments, Spain) for 1minute and then incubated at 37°C for 18–24h. The same microbiological procedure was followed for water samples after removing the full gauzes from the cassettes and placed them in peptone water. After incubation, two plates with MacConkey agar medium (BD) were inoculated with 100μl from each stomacher bag; one plate was supplemented with 2μg/ml of ceftazidime (CAZ; Sigma, Germany) and the other with 2μg/ml of ciprofloxacin (CIP; Sigma, Germany). In all experiments, *K. pneumoniae* SCL 2346 (CAZ MIC >16μg/ml and CIP MIC >2μg/ml) and *E. coli* ATCC 25922 were used as resistant and susceptible controls, respectively. All plates were incubated at 37°C for 18–24h. Colonies were selected according to morphology, using a magnifying glass. This selection was made by classifying the colonies according to standard patterns: colony shape, color (pigmentation), texture, and edge shape, as described previously (Higuera-Llantén et al., 2018). Distinct morphotypes phenotypically consistent with Enterobacterales were further identified by MALDI-TOF (Bruker Daltonics, Germany).

### Antimicrobial Susceptibility Testing

All isolates confirmed as Enterobacterales were tested against a panel of 15 antibiotics using the disk diffusion method following CLSI guidelines (CLSI, Clinical and Laboratory Standards Institute, 2018). Antibiotic tested included as ampicillin (AMP, 10μg); cefazolin (CFZ, 30μg); ceftazidime (CAZ, 30μg); ceftriaxone (CRO, 30μg); cefepime (FEP, 30μg); ertapenem (ETP, 10μg); imipenem (IPM, 10μg); meropenem (MEM, 10μg); ampicillin/sulbactam (SAM, 10/10μg); piperacillin/tazobactam (TZP, 100/10μg); ciprofloxacin (CIP, 5μg); amikacin (AMK, 30μg); gentamicin (GEN, 10μg); fosfomycin/trometamol (FOF, 200μg); and trimethoprim/sulfamethoxazole (SXT, 1.25/23.75μg), all of which were supplied by OXOID (Hampshire, England). Bacterial isolates resistant to three or more antimicrobial classes were cataloged as MDR following previously standardized criteria (Magiorakos et al., 2012). Intrinsic resistance was not considered for susceptibility analysis in *Klebsiella aerogenes* (CFZ and SAM), *Citrobacter freundii* (AMP, CFZ, and SAM), and *Enterobacter cloacae* (CFZ). Importantly, all *Citrobacter* spp. were analyzed as members of the *C. freundii* complex due to the impossibility of identification down to the species level using the MALDI-TOF technique (Kolínská et al., 2015). Isolates classified as intermediate were considered resistant bacteria for the purpose of this study.

### Molecular Detection of Extended Spectrum β-lactamases and *mcr-1*

Isolates exhibiting resistance to third- or fourth-generation cephalosporins (CAZ, CRO, and FEP) or carbapenems (ETP and IMP) were tested to detect the presence of genes encoding ESBLs (*bla*_TEM_, *bla*_SHV_, and *bla*_CTX-M_) by a multiplex PCR scheme as previously reported (Salgado-Caxito et al., 2021).

To test a possible resistance to colistin in all isolates, a PCR to detect the *mcr-1* gene was performed. Primers used were previously reported (Rebelo et al., 2018). Briefly, running conditions were as: 1cycle of denaturation at 95°C for 15min, followed by 10cycles of extension: 30s at 95°C, 30s at 58°C, and 1min at 72°C. The final cycle of elongation was performed for 5min at 72°C. The amplification was visualized by electrophoresis using 2% agarose. *E. coli* isolate SCL1290 (mcr-1 positive) was used as the positive control.

### Statistical Analysis

Antimicrobial resistance profiles were compared among sources by a clustering and a heat map. These represented each isolate resistance/no resistance to previously described antibiotics. Analysis was conducted using Heat-map.plus in R (version 4.0.0). The heat map also included the antibiotic used for isolation, season of isolation collection, bacterial species, source of isolation, and MDR profile (yes/no).

We used generalized linear models (GLM) to explore possible associations between the environmental variables registered during each sampling effort and isolation of MDR Enterobacterales, evaluating each matrix separately. The multivariate statistical model for vegetable samples included all explanatory variables and was selected as the best model based on the Akaike information criterion (AIC=142.11) compared to models including only one variable (AIC>216.49). The statistical significance (*p*<0.05) of each variable in the full model was assessed using Wald’s test. As an insufficient number of Enterobacterales was isolated from water samples, GLM could not be conducted. Instead, boxplot (R) or histograms (Excel) were constructed to display the distribution of the data based on isolation of MDR Enterobacterales considering different environmental factors. Some environmental factors were not recorded during the first vegetable sampling (produce condition, wearing gloves while manipulating vegetables, presence of pets, and presence of pest control devices), these were not included in the statistical analysis. Drinking water was available and used in all the farmer markets studied, as well as produce separation by type, therefore, these variables were also not included in the GLM analysis.

It was evaluated the association between each Enterobacterales isolate presenting MDR as defined above (yes/no), with two independent categorical variables (source of the sample and season) that were present in both the vegetable and river dataset: (i) source in which the sample was collected, including “river,” “beet,” “celery,” chard,” and “parsley”, except for “coriander” that had to be removed from the analysis for having only one sample in which MDR Enterobacterales was isolated, which meant the analysis could not be run; (ii) season in which the sample was collected, with “rainy” including winter and fall and “dry” including spring and summer; and (iii) the variable “CIP/CAZ selection” corresponding to the antibiotic used as a supplement in the MacConkey agar to isolate clinically relevant Enterobacterales (CIP/CAZ). A similar analysis was conducted categorizing each Enterobacterales isolate resistant to antibiotics of different families: (i) resistant to 3GC (CAZ and/or CRO); (ii) resistant to penicillin with β-lactamase inhibitor (RPIB) antibiotics (SAM and/or TZP); (iii) resistant to the quinolone CIP (RCIP); and (iv) resistant to β-lactam (RBET) antibiotics (AMP, ETP, IPM, MEM, CAZ, CRO, CFZ, FEP, SAM, and/or TZP). However, in these models, the variable “CIP/CAZ selection” was not included. Resistance (yes/no) was characterized as described above based on the CLSI standards.

The binary nature of the response variable (yes/no) required the use of generalized linear models (GLM) with binomial errors (such as logistic regression), including the two variables (source and season) listed above. Analyses of the generalized linear model were performed using the R software^1^ (version 4.0.0) The multivariate statistical model included all explanatory variables and was selected as the best model based on the Akaike information criterion (AIC=189.16 for the MDR outcome, AIC=214.51 for the 3GC outcome, AIC=177.77 for the RPIB outcome, AIC=171 for the RCIP outcome, and AIC=190.07 for the RBET outcome) compared to models including only one variable (AIC>216.49). The statistical significance (*p*<0.05) of each variable in the full model was assessed using Wald’s test.

GLM could not be performed for the models to evaluate the association between each Enterobacterales isolates selected using CIP and CAZ resistant to: (i) at least one antibiotic, regardless of their family, as all the isolates were resistant to at least one of the antibiotics evaluated in the study; (ii) carbapenems (ETP, IPM, and/or MEM); and (iii) aminoglycosides (AMK and/or GEN). The GLM for the carbapenem and aminoglycoside outcomes could not be performed because all the Enterobacterales isolates recovered from beets (*n*=9) were susceptible to carbapenems and aminoglycoside antibiotics as per the CLSI standards.

## Results

### Prevalence of Resistant Enterobacterales in Vegetables

CAZ- and CIP-resistant Enterobacterales were obtained from vegetables during the four samplings. Isolates showed an overall prevalence of 8.8% (42/478) and 17.5% (84/478) for those selected with CAZ or CIP, respectively. For CAZ resistance, the highest prevalence of 35.7% (10/28) and 30.6% (11/36) was found for samples from the *Apiaceae* family (i.e., parsley, coriander, and celery) in the spring and summer, respectively. This was followed by chard samples, which presented a prevalence of 27.6% (8/29) in the summer and 12.5% (3/24) in the spring. Beet and lettuce, also during the summer, showed the higher prevalence (Table 1). Interestingly, all 120 vegetable samples obtained during the winter were negative for CAZ-resistant Enterobacterales.

**Table 1:** Prevalence of Enterobacterales detected with ceftazidime and ciprofloxacin added plates on vegetable and river samples.

	Percentage of positive samples^2^ (Number of positive samples/total samples) in a given matrix obtained on plates with CAZ and CIP									
Season^1^	Parsley, Coriander, and Celery^3^		Lettuce		Beet		Chard		Water^4^	
	CAZ	CIP	CAZ	CIP	CAZ	CIP	CAZ	CIP	CAZ	CIP
Fall	6.7% (2/30)	73.3% (22/30)	0% (0/30)	10% (3/30)	0% (0/30)	13.3% (4/30)	0% (0/30)	30% (9/30)	37.5% (3/8)	37.5% (3/8)
Winter	0% (0/30)	0% (0/30)	0% (0/35)	0% (0/35)	0% (0/30)	0% (0/30)	0% (0/25)	0% (0/25)	0% (0/8)	25% (2/8)
Spring	35.7% (10/28)	50% (14/28)	2.8% (1/36)	8.3% (3/36)	3.3% (1/30)	0% (0/30)	12.5% (3/24)	8.3% (2/24)	0% (0/8)	0% (0/8)
Summer	30.6% (11/36)	44.4% (16/36)	9.7% (3/31)	6.5% (2/31)	12.5% (3/24)	4.2% (1/24)	27.6% (8/29)	27.6% (8/29)	0% (0/8)	37.5% (3/8)
Total	18.5% (23/124)	41.9% (52/124)	3% (4/132)	6.1% (8/132)	3.5% (4/114)	4.4% (5/114)	10.2% (11/108)	17.6% (19/108)	9.4% (3/32)	25% (8/32)

1 For all seasons, five farmer markets were sampled.

2 Included samples with at least one isolate recovered per vegetable and water samples. Total of vegetable samples was 478 and total of river samples was 32.

3 These three vegetables were grouped together since they belong to the same family of *Apiaceae*.

4 Water samples included samples of Lontue and Claro rivers.

CIP, ciprofloxacin; CAZ, ceftazidime.

The highest prevalence of CIP-resistance was found in samples from *Apiaceae* during the fall [73.3% (22/30)], spring [50% (14/28)], and summer [44.4% (16/36)]. In chard samples, we observed a prevalence of 30% (9/30) for the fall, 8.3% (2/24) for the spring, and 27.6% (8/29) for the summer. Lettuce samples showed a prevalence of 10% (3/30), 8.3% (3/36), and 6.5% (2/31) for the fall, spring, and summer, respectively. Finally, beet samples were positive only during the fall [13.3% (4/30)] and the summer [4.2% (1/24)] (Table 1). Similar to CAZ-resistant Enterobacterales, none of the 120 vegetable samples obtained during the winter season were positive.

### Prevalence of Resistant Enterobacterales Isolated From Water

For the prevalence of isolates obtained in CAZ contained plates, the highest prevalence of 37.5% (3/8) was observed in water samples during the fall (Table 1); however, in the water samples, none of the tested samples were positive for CAZ-resistant Enterobacterales during the other seasons. On the other hand, CIP-resistant Enterobacterales were observed in three seasons, with a prevalence of 37.5% (3/8) for the fall and summer and a prevalence of 25% (2/8) in the winter (Table 1).

### Identification and Distribution of Antibiotic-Resistant Enterobacterales Species in Vegetables and Water

A total of 155 ARB was confirmed as Enterobacterales. The predominant species in vegetables and river samples was *E. coli*, with 70.3% (109/155) of the isolates, which were obtained from the *Apiaceae* family (*n*=57 strains), chard (*n*=18), water samples (*n*=17), lettuces (*n*=10), and beets (*n*=7; Table 2). Moreover, *Citrobacter* spp. was the second most common (18.7%, 29/155) and was found in the *Apiaceae* family (*n*=17), chards (*n*=7), beets (*n*=2), and lettuces (*n*=2); one isolate was recovered from a water sample. Nine isolates belonging to the genus *Klebsiella* spp. were found (5.8%, 9/155), corresponding to only two species: *K. pneumoniae* and *K. aerogenes*; while the first one was found in the *Apiaceae* family, and in chards and water, *K. aerogenes* was found only once in a parsley. Finally, *E. cloacae* complex (2.5%, 8/155) was detected in parsleys, a lettuce, and a chard (Table 2). In addition to the Enterobacterales order, other isolates were identified, including *Aeromonas* sp., which was recovered from vegetables collected in both rainy and dry seasons (data not shown).

**Table 2:** Identification and distribution of Enterobacterales strains isolated from vegetable and river samples collected during four seasons during 2019–2020.

	Number of isolates obtained					
Species	Parsley, Coriander, and Celery^1^	Lettuce	Beet	Chard	Water^3^	Total
*Escherichia coli*	57^2^	10	7	18	17^b^	109
*Citrobacter sp*	17^2^	2	2	7	1	29
*Klebsiella pneumoniae*	2	0	0	5^2^	1	8
*Klebsiella aerogenes*	1	0	0	0	0	1
*Enterobacter cloacae* complex	6^2^	1	0	1	0	8
Total	83	13	9	31	19	155

1 These three vegetables were grouped together since they belong to the same family of *Apiaceae*.

2 More than one isolate was obtained from the same sample.

3 Water samples included samples of Lontue and Claro rivers.

### Antimicrobial Resistance Profile in Isolates Recovered From Vegetables and Water

Considering the 155 bacterial isolates from vegetables and water, the susceptibility test showed that the highest resistance was to AMP and CIP, with 63.2% and 74.2%, respectively. Resistance to cephalosporins was observed in CFZ, which presented 38.5% of resistance, CAZ 34.8%, CRO 34.2%, and FEP 16.1%. The carbapenems ETP and IPM showed 13.5% and 1.9% of resistance, respectively, while no MEM resistant isolates were obtained during the study. For β-lactams + β-lactamase inhibitor, SAM presented 24.8% of resistance and TZP 8.4%. For aminoglycoside resistance, AMK and GEN showed 1.9% and 3.2%, respectively. For FOF, 2.6% of the strains presented resistance. Finally, for SXT, 41.9% showed resistance (Supplementary Table S1).

**Figure 2:**
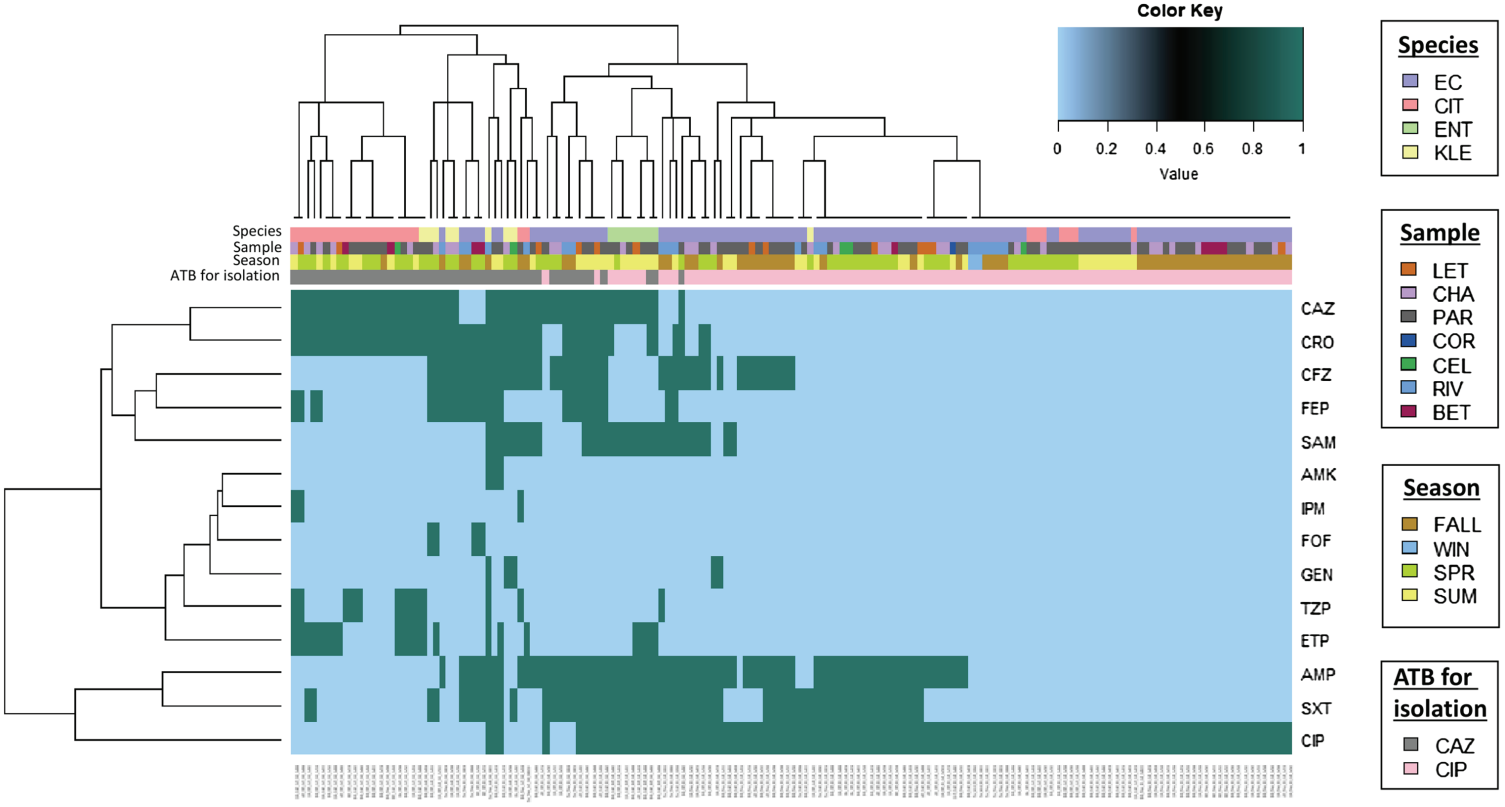
Heat map representation of antimicrobial resistance (AMR) profiles. Cluster analysis of AMR profiles, in the vertical axis the abbreviations for the antimicrobial tested and in the horizontal axis are the isolates obtained in this study. Light blue represents sensitivity and dark green represents resistance. Five clusters were found and are labeled from 1 to 5 in the dendrogram.

To analyze similarity among antibiotic resistance patterns and sources, a clustering was performed (Figure 2). Five main clusters were identified as: Cluster 1 grouped 21 CAZ-selected isolates [*Citrobacter* spp. (*n*=20) from parsley, chard, lettuce, beet, and celery and *K. aerogenes* (*n*=1) from parsley] (Figure 2). Nine isolates in cluster 1 presented an MDR phenotype. Cluster 2 grouped 18 isolates (three *E. coli* from water, six *E. coli* from parsley, beet, and lettuce, two *Citrobacter* spp. from parsley and water, and seven *K. pneumoniae* from parsley, chard, celery, and water), all selected in CAZ from vegetables collected in the fall, spring, and summer and in water samples collected during the fall. All isolates except two *K. pneumoniae* from chards presented an MDR profile. Cluster 3 grouped 26 MDR isolates (five *E. coli* from water, 13 *E. coli* from parsley, chard, and lettuce, and eight *E. cloacae* complex from parsley, chard, and lettuce) selected in CAZ and CIP from vegetables collected in three seasons (fall, spring, and summer) and from water collected in the fall and summer. Cluster 4 grouped 40 isolates (39 *E. coli* isolated from parsley, lettuce, chard, beet, celery, coriander, and water and one *K. pneumoniae* isolated from a chard), all selected in CIP and included isolates from vegetables collected in the fall, spring, and summer, along with isolates from water collected in the fall and summer. A total of 29 *E. coli* presented MDR. Finally, cluster 5 grouped 50 isolates (43 *E. coli* isolated from parsley, chard, beet, lettuce, celery, and water and seven *Citrobacter* spp. isolated from parsley and chard) that were selected and resistant only to CIP; these isolates were obtained from vegetables collected in the fall, spring, and summer, and from samples from water collected in the fall and winter. None of the isolates in cluster 5 presented MDR.

### Presence of *mcr-1* and *bla*_ESBL_ Genes in Isolates From Vegetables and Water

The gene *mcr-1* was found in 2/155 isolates; both isolates corresponded to *E. coli* that were isolated during the summer season, from two distinct beet samples from the same market. These two *E. coli* isolates presented identical antibiotic resistance patterns (Table 3). Detection of ESBL genes was performed in 62/155 isolates that presented resistance to third-generation cephalosporins and/or carbapenems. ESBL genes were found in 23/62 isolates tested, representing seven isolates of *K. pneumoniae* (11.3%) and 16 isolates of *E. coli* (25.8%; Table 3). The most common ESBL gene detected was *bla*_CTX-M_ found in *E. coli* (*n*=15) and *K. pneumoniae* (*n*=5), followed by *bla*_SHV_ found in *K. pneumoniae* (*n*=5). Finally, *bla*_TEM_ was detected in *E. coli* (*n*=3) and *K. pneumoniae* (*n*=1). Combinations of *bla*_CTX-M_, *bla*_SHV_ and *bla*_CTX-M_, *bla*_TEM_ were also found. Summer was the season with more isolates containing ESBL genes, with 10/23 isolates; no ESBL genes were detected in the isolates collected during the winter.

**Table 3:** Resistance profile and presence of *mcr-1* and *bla*ESBL genes on *E. coli* and *K. pneumoniae* isolates.

Species	Source	Season	Resistance Profile^2^	*bla* genes or *mcr*
*E. coli*	Parsley	Spring	AMP-CFZ-CAZ-CRO-FEP-ETP-SAM-CIP-AMK-SXT	*bla*_CTX-M_
*E. coli*	Parsley	Spring	AMP-CFZ-CAZ-CRO-FEP-SAM-CIP-AMK-SXT	*bla*_CTX-M_
*E. coli*	Lettuce	Spring	AMP-CFZ-CAZ-CRO-FEP-SAM-CIP-SXT	*bla*_CTX-M_
*E. coli*	Parsley	Spring	AMP-CFZ-CAZ-CRO-FEP-SAM-CIP-SXT	*bla*_CTX-M_
*E. coli*	Parsley	Spring	AMP-CFZ-CAZ-CRO-FEP-SAM-CIP-SXT	*bla*_CTX-M_
*E. coli*	Parsley	Spring	AMP-CFZ-CAZ-CRO-FEP-SAM-CIP-SXT	*bla*_CTX-M_
*E. coli*	Lettuce	Spring	AMP-CFZ-CAZ-CRO-FEP-CIP-SXT	*bla*_CTX-M_
*E. coli*	River	Summer	AMP-CFZ-CRO-FEP-SAM-CIP-SXT	*bla*_CTX-M_
*E. coli*	Beet	Summer	AMP-CFZ-CRO-FEP-FOF-SXT	*bla*_CTX-M_, *bla*_TEM_, *mcr-1*
*E. coli*	Beet	Summer	AMP-CFZ-CRO-FEP-FOF-SXT	*bla*_CTX-M_, *bla*_TEM_, *mcr-1*
*E. coli*	Parsley	Summer	AMP-CAZ-CIP-SXT	*bla*_TEM_
*E. coli*	River	Fall	AMP-CFZ-CAZ-FEP-CRO-SXT	*bla*_CTX-M_
*E. coli*	River	Fall	AMP-CFZ-CAZ-FEP-CRO-SXT	*bla*_CTX-M_
*E. coli*	River	Fall	AMP-CFZ -CAZ-FEP-CRO	*bla*_CTX-M_
*E. coli*	River	Fall	AMP-CFZ-FEP-CRO-SXT	*bla*_CTX-M_
*E. coli*	River	Fall	AMP-CFZ-FEP-CRO-SXT	*bla*_CTX-M_
*K. pneumoniae*	Chard	Summer	CFZ-CAZ-CRO-FEP-ETP-FOF-SXT	*bla*_CTX-M,_ *bla*_SHV_
*K. pneumoniae*	Celery	Summer	CFZ-CAZ-CRO-SAM-GEN-SXT	*bla*_SHV_
*K. pneumoniae*	Parsley	Summer	CFZ-CAZ-CRO-FEP-FOF-SXT	*bla*_CTX-M_, *bla*_SHV_
*K. pneumoniae*	Chard	Summer	CFZ-CAZ-CRO-SAM-GEN	SHV
*K. pneumoniae* ^1^	Chard	Summer	CFZ-CAZ-CRO-FEP	*bla*_CTX-M_, *bla*_SHV_
*K. pneumoniae* ^1^	Chard	Summer	CFZ-CAZ-CRO-FEP	*bla*_CTX-M_, *bla*_SHV_
*K. pneumoniae*	River	Fall	AMP-CFZ -CAZ-CRO-FEP-ETP-SAM-CIP-GEN-SXT-TZP-AMK	*bla*_CTX-M_, *bla*_TEM_

1 Isolates resistant only to cephalosporins.

2 AMP, ampicillin; CFZ, cefazolin; CAZ, ceftazidime; CRO, ceftriaxone; FEP, cefepime; ETP, ertapenem; IPM, imipenem; MEM, meropenem; SAM, ampicillin/sulbactam; TZP, piperacillin/tazobactam; CIP, ciprofloxacin; AMK, amikacin; GEN, gentamicin; FOF, fosfomycin/trometamol; and SXT, trimethoprim/sulfamethoxazole.

### Descriptive Evaluation of Environmental Factors and MDR Enterobacterales Isolation in River Water Samples

The results of the descriptive statistics of isolation of MDR Enterobacterales in water samples based on continuous environmental factors registered when samples were collected are shown in Supplementary Figure S2. In water samples, 50% of the MDR Enterobacterales isolated in river water were recovered in water with the following characteristics: pH ranging between 6.1 and 7.7, temperature between 5°C and 13°C, conductivity between 100μs and 250μs, total dissolved solids between 20 and 160ppm; salinity between 20ppm and 120ppm; and an ambient temperature between 1°C and 14°C (Supplementary Figure S2).

The descriptive statistics conducted for the categorical environmental factor (Supplementary Figure S3) show that MDR Enterobacterales were (i) more frequently isolated from the Claro river in the rainy season and (ii) slightly more frequent using CAZ instead of CIP as a supplement in the MacConkey agar, (iii) in sunny weather conditions, (iv) in sampling sites in which there was no observation of crops nearby, (v) in sites where a rain event occurred 5days prior to the water sample collection, (vi) in sites where at least one animal was present when the sample was collected, and (vii) in sites where aquatic plants were present in the sampling point.

### Association Between Environmental Factors and MDR Enterobacterales Isolation in Vegetable Samples

The descriptive statistics conducted for the categorical and continuous environmental factors (Supplementary Figure S4) show that MDR Enterobacterales detected in vegetables were (i) more frequently isolated from parsley and chard, (ii) during the dry season, (iii) in sites where a rain event did not occurred 5days prior to the vegetable sample collection, (iv) in partly cloudy weather conditions, (v) slightly more frequent using CAZ instead of CIP as a supplement in the MacConkey agar, and (iv) in ambient temperature ranging from 18°C to 27°C approximately.

The generalized linear model presented in Table 4 shows that Enterobacterales that grew in MacConkey agar supplemented with CAZ were statistically significantly associated with the isolation of MDR Enterobacterales in vegetable samples, with CAZ used as supplement having a 3.1 times higher likelihood of isolating MDR Enterobacterales compared to those that were isolated in plates supplemented with CIP (OR=0,073; 95% CI: [1.217, 8.2623]; *p*<0.021). All the other environmental factors (produce type, season, produce state, insect in farmers market, and produce at ground level) presented a non-significant association with MDR Enterobacterales in the GLM model (value of *p* >0.05).

**Table 4:** Multivariable generalized linear model showing risk factor associations between environmental factors and isolation of MDR Enterobacterales strains isolated from vegetables collected in November 2019 and January 2020.

Variable	Categories or levels^1^	Odds ratio estimates^2^	SD Error	95% CI limits	*P*
Produce	Parsley^3^				
	Beet	0.802	1.0731	(0.096, 7.718)	0.837
	Celery	3.596	1.2200	(0.415, 78.524)	0.294
	Chard	0.698	0.5888	(0.215, 2.205)	0.541
	Lettuce	1.183	0.8666	(0.220, 7.200)	0.846
Season^4^	Summer^3^				
	Spring	1.913	0.9730	(0.298, 16.139)	0.505
Produce State	Fresh^3^				
	Partly Rotten	2.927	0.7116	(0.764, 13.104)	0.131
	Completely Rotten	0.437	1.1446	(0.039, 3.981)	0.470
Insects in farmers market	Yes^3^				
	No	0.3969	1.0318	(0.0495, 2.965)	0.371
Produce at ground level	Yes^3^				
	No	0.498	0.7339	(0.112, 2.123)	0.342
Enterobacterales isolation with CAZ or CIP	CIP^3^				
	CAZ	3.073	0.4854	(1.217, 8.2623)	0.021^5^

1 Vegetable samples with Enterobacterale*s* strains isolated in MacConkey supplemented with 2μg/ml of ciprofloxacin (CIP) and 2μg/ml of ceftazidime (CAZ).

2 AIC=142.11; R-sqr=0.1091288.

3 Used as the reference category for statistical comparisons.

4 Spring: September 21st to Dec 20th 2021; Summer: Dec 21st to March 20th.

5 Risk factors with statistically significant results.

### Association Between the Environmental Factors Season and Source of the Sample, and AMR Enterobacterales Isolation in Both Vegetable and Water Samples

The seasonality analyses shown in Table 5 indicate that Enterobacterales isolates recovered from vegetable samples collected during the rainy season were significantly less likely to be resistant to at least one 3GC (OR=0.08; 95% CI: [0.023–0.227]; *p*<0.00001) compared to those recovered in samples collected in the dry season (Table 5). Similarly, Enterobacterales isolates recovered from samples collected during the rainy season were significantly less likely to be resistant to at least one penicillin combined with a β-lactamase inhibitor (OR=0.18; 95% CI: [0.05–0.47]; *p*<0.0015) and to at least one β-lactam antibiotic (OR=0.22; 95% CI: [0.098–0.482]; *p*<0.0001) compared to Enterobacterales isolates recovered from samples collected during the dry season (Table 5). In contrast, Enterobacterales previously isolated from samples collected during the rainy season were 5.12 times more likely to be resistant to CIP in the disk diffusion method than isolates recovered in samples collected during the rainy season (OR=5.109; 95% CI: [1.069, 19.806]; *p*=0.0034).

**Table 5:** Multivariate generalized linear model testing the influence of Matrix and season on the likelihood of each Enterobacterales isolate’s resistance based on Kirby-Bauer and CLSI standards: **(A)** Multidrug resistance (MDR)^1^ and resistance to at least one antibiotic of the following families: **(B)** third-generation cephalosporin (3GC)^2^; **(C)** Penicillin with β-lactamase inhibitor (RPIB)^3^; **(D)** Ciprofloxacin (RCIP)^4^; and **(E)** β-lactam (RBET)^5^.

Model	Variable	Level	OR	OR 95%CI	Std. error	*z* value	Pr (>|z|)
A. MDR^1^	Matrix^6^^,^^7^	Beet	0.243	(0.035, 1.421)	0.93	−1.521	0.1283
		Celery	1.960	(0.194, 46.447)	1.29	0.522	0.6017
		Chard	0.450	(0.119, 1.636)	0.66	−1.205	0.2281
		Lettuce	1.399	(0.280, 7.550)	0.82	0.406	0.6849
		Parsley	0.601	(0.185, 1.901)	0.58	−0.866	0.3865
	Season^8^^,^^9^	Rainy	0.555	(0.245, 1.224)	0.40	−1.444	0.1487
B. 3GC^2^	CIP/CAZ Selection^10^		3.282	(1.519, 7.420)	0.40	2.953	0.0032^11^
	Matrix^6^^,^^7^	Beet	0.411	(0.053, 2.862)	1.00	−0.883	0.3774
		Celery	0.129	(0.011, 1.235)	1.17	−1.752	0.0797
		Chard	0.203	(0.039, 0.930)	0.80	−1.992	0.0464^11^
		Lettuce	0.256	(0.039, 1.508)	0.92	−1.476	0.1399
		Parsley	0.201	(0.043, 0.814)	0.73	−2.178	0.0294^11^
	Season^8^^,^^9^	Rainy	0.082	(0.023, 0.226)	1.00	−4.386	<0.0001^11^
C. RPIB^3^	Matrix^6^^,^^7^	Beet	0.189	(0.008, 1.822)	1.28	−1.305	0.1919
		Celery	0.604	(0.054, 5.955)	1.16	−0.433	0.6642
		Chard	0.371	(0.072, 1.910)	0.81	−1.208	0.2270
		Lettuce	0.536	(0.082, 3.338)	0.93	−0.669	0.5033
		Parsley	0.616	(0.146, 2.722)	0.73	−0.667	0.5046
	Season^8^^,^^9^	Rainy	0.174	(0.053, 0.472)	0.55	−3.179	0.0014^11^
D. RCIP^4^	Matrix^6^^,^^7^	Beet	0.896	(0.170, 2.86)	0.94	−0.117	0.9066
		Celery	2.115	(0.141, 5.958)	1.15	0.651	0.5153
		Chard	1.761	(0.222, 23.604)	0.76	0.745	0.4560
		Lettuce	3.647	(0.394, 8.173)	0.95	1.359	0.1740
		Parsley	4.513	(0.595, 26.576)	0.73	2.067	0.0387^11^
	Season^8^^,^^9^	Rainy	5.109	(1.069, 19.806)	0.56	2.929	0.0034^11^
E. RBET^5^	Matrix^6^^,^^7^	Beet	0.431	(0.072, 2.523)	0.89	−0.945	0.3448
		Celery	0.689	(0.070, 16.050)	1.28	−0.291	0.7707
		Chard	0.509	(0.131, 1.889)	0.68	−0.999	0.3180
		Lettuce	3.557	(0.462, 75.096)	1.19	1.070	0.2847
		Parsley	0.475	(0.140, 1.502)	0.60	−1.243	0.2140
	Season^8^^,^^9^	Rainy	0.221	(0.098, 0.482)	0.40	−3.726	0.0002^11^

1 AIC=189.16; *r*2=0.07131851.

2 AIC=214.51; *r*2=0.1678267.

3 AIC=177.77; *r*2=0.0984252.

4 AIC=171; *r*2=0.107578.

5 AIC=190.07; *r*2=0.1302794.

6 For the variable Matrix, the level “coriander” had to be removed from the model because this food had only one Enterobacterales isolate, which did not present MDR, therefore the GLM presented error.

7 Water sample was used as the reference for the variable Matrix to estimate the effects of each variable category.

8 Dry was used as the reference for the variable Matrix to estimate the effects of each variable category.

9 Season in which the sample was collected with “Rainy” representing March 21st to September 20th, and “Dry” from September 21st to March 20th for a Mediterranean climate in the Southern Hemisphere of the American continent.

10 CIP/CAZ selection corresponds the antibiotic used to select Enterobacterales AMR isolates. CIP was used as the reference for the variable CIP/CAZ selection to estimate the effects of each variable category.

11 Variables with statistical significance (*p*<0.05).

For the variable matrix, the statistical analyses showed that Enterobacterales isolated from chard and parsley were 0.2 times less likely to be resistant to at least one 3GC compared to the isolates obtained from river (OR=0.203 [95% CI: 0.038, 0.930]; *p*=0.046 and OR=0.202 [95% CI: 0.043, 0.814]; *p*<0.029, respectively; Table 5). Similarly, Enterobacterales isolated from parsley were 4.51 more likely to be resistant to CIP compared to those isolated from the river ([95% CI: 0.5945633, 26.576365]; *p*=0.039).

The generalized linear model showed that Enterobacterales that grew in MacConkey agar supplemented with CAZ were the only statistically significant variable associated with the isolation of MDR Enterobacterales (Table 5); this indicates that Enterobacterales that grew in CAZ supplemented plates were 3.28 more likely to be MDR Enterobacterales compared to those that grew in CIP supplemented plates (OR=3.28 [95% CI: 1.519, 7.421] value of p: 0.003). All the other variables included in the model were not significantly associated with the isolation of MDR Enterobacterales (value of *p* >0.05).

## Discussion

In this study, antibiotic-resistant Enterobacterales were recovered and characterized from vegetables and water samples in an agricultural city in central Chile; variation in seasons and source composition were analyzed to search for associations with the presence of resistant and MDR Enterobacterales. The main findings of this work are as: (i) wide distribution and significant prevalence of resistant and clinically relevant Enterobacterales in both vegetables and surface water; (ii) colistin resistance gene *mcr-1* and ESBL coding genes found in isolates obtained from vegetables and water; and (iii) association of seasons of the year with the isolation of MDR and third-generation cephalosporin resistant Enterobacterales in vegetables and water samples.

### Wide Distribution and Significant Prevalence of Resistant and Clinically Relevant Enterobacterales in Vegetables and Water

Currently, there is scarce information about AMR in vegetables and the risk of ARB acquisition by consumption of contaminated vegetables; in this work, we found a high prevalence of resistant and MDR Enterobacterales collected from vegetables, mostly in sampled parsleys, corianders, and celeries. While many food safety research has focused on well-known foodborne pathogens, such as *Salmonella*, *Listeria monocytogenes*, and *E. coli* O157-H7 in vegetables (Rajabzadeh et al., 2018), our study screened for antibiotic-resistant Enterobacterales. In other studies, distinct prevalence of resistant Enterobacterales has been reported in food, for instance, Richter et al., 2019 found that spinaches and tomatoes, obtained at different points of sale (grocery stores, vendors, and farmer markets), were the most frequently contaminated, with a prevalence of 17.4%, which was similar to our study. However, a much lower prevalence (2.7%) was reported for 3GC resistance bacteria in supermarket vegetables in Netherlands (Blaak et al., 2014). This highlights the relevance of understanding local level of contamination and the public health implications of the consumption of ready-to-eat vegetables contaminated with resistant Enterobacterales.

In this study, we also investigated resistant Enterobacterales in water samples; here, we found an important prevalence of resistance and MDR Enterobacterales. Likewise vegetable samples, different prevalence results have been reported, as a similar study that reported highly contaminated water samples (15/22 samples) collected in estuaries in the Lebanon (Diab et al., 2018), where 45% of these isolates presented an MDR profile (*E. coli, K. pneumoniae*, and *Citrobacter* spp). These results suggest that rivers used to irrigate the farms, carry antimicrobial-resistant Enterobacterales, and could be a potential source of contamination for the vegetables that were locally produced and consumed throughout the year (Jongman and Korsten, 2016; Hudson et al., 2017). While traceability of sampled vegetables was not possible with our approach (sampling at farmer markets), rather than at the harvesting sites, further whole genome sequencing could facilitate to elucidate closeness of resistant Enterobacterales obtained from water and vegetable samples.

In this work, we not only found high prevalence, but also presence of clinically relevant Enterobacterales (3GC resistant *E. cloacae* complex and *E. coli,* and *K. pneumoniae*). *K. pneumoniae* and *E. cloacae* complex are considered opportunistic pathogens, and several reports indicated that they are associated with important hospital-acquired infections (De Oliveira et al., 2020). Other similar studies have also reported *Enterobacter* and *Klebsiella* genera in fresh vegetables; more concerning, they found a higher concentration of *E. cloacae* in ready-to-eat food products (Falomir et al., 2013). Our findings along with previous studies are extremely interesting because it creates the possibility of knowing the potential risk of acquiring clinically relevant bacteria from food. These bacteria could colonize the gut microbiota of people or could even cause a higher risk of developing an infection in immunocompromised people. Although the evidence is strong, more studies are needed to trace these bacteria and their fate after people ingest them.

### Colistin Resistance Gene *mcr-1* and ESBL Were Found in Isolates Obtained From Vegetables and Water

In this publication, two *E. coli* isolates that carry the *mcr-1* gene were found. This result represents the first report of colistin-resistant bacteria in vegetables in Latin America and the first time that the *mcr-1* gene has been described in non-clinical environments in Chile. Nowadays, colistin is considered “last-line therapy” to treat infections caused by MDR-resistant Gram-negative bacteria, such as *E. coli* and *K. pneumoniae* (Jones et al., 2013) and there is an increasing worldwide interest in bacteria isolated from vegetables carrying the *mcr-1* gene due to the potential risk of acquiring this gene through food consumption. Colistin-resistant bacteria isolated from vegetables and carrying some alleles of the *mcr-1* gene have already been described in diverse other countries (Zurfuh et al., 2016; Liu et al., 2020; Manageiro et al., 2020), which constitutes a major public health problem. Importantly, the two isolates that carried *mcr-1* genes found in this study, also carried the β-lactamases bla_CTX-M_ and *bla*_TEM_. Although this work did not study the genetic context of these genes, it would be interesting to evaluate it in a future study since it has been described that the *mcr-1* gene with different β-lactamases can coexist in the same plasmid that are highly transmissible (Migura-Garcia et al., 2020).

Our work found that ESBL had a prevalence of 11.3% in *K. pneumoniae* and 25.8% in *E. coli.* Similar results were presented in a previous study that collected 109 vegetable samples from 18 farms in Tunisia and detected ESBL genes in isolates of *E. coli*, *Klebsiella*, and *Citrobacter*; likewise our study, *bla*_CTX-M_ was the most common ESBL detected, and *bla*_TEM_ and *bla*_SHV_ were also found (Said et al., 2015). Additionally, ESBL genes have also been reported from different water sources, Caltagirone et al., 2017 conducted a study in Italy and reported the presence of *bla*_CTX-M_ along with *bla*_CTX−M_ in combination with *bla*_SHV_ in isolates obtained from wells, streams, and water treatment plants. In Chile, a recent study reported the prevalence of ESBL in *E. coli* in domestic and wild animals, indicating a 30% prevalence in livestock, 24% in dogs, and 0.5% in wild animals; meanwhile, CTX-M enzymes were the most common enzymes in this study, coinciding with the results reported in the present work (Benavides et al., 2021). Other studies in the country have reported *bla*_CTX-M_ in foxes, Andean condors, and wild felids (Cevidanes et al., 2020; Sacristán et al., 2020). These results highlight a high incidence of *bla*_CTX-M_ in different environments in Chile and the fact that run-off from domestic animals could be a source of contamination for water and food. Significantly, AMR should be studied with a focus on one health because *bla*_CTX-M_ was found to present a high prevalence in an intensive care unit in the country (Pavez et al., 2019). The present study, along with previously reported studies, highlights the relevance of improving our understanding of the environmental dissemination of ESBL in water sources and vegetables. Further studies are necessary, including whole genome sequencing of the collected isolated harboring ESBL, to understand at the genomic level the isolates and mobile genetic elements that are involved in the dispersion and transmission of these genes.

### Seasons Is Associated With the Isolation of MDR and Third-Generation Cephalosporins Enterobacterales in Vegetables and Water

Statistical analysis performed in this work indicated that the rainy season (fall and winter) has a lower likelihood of isolating Enterobacterales that displayed an MDR phenotype and resistance to at least one 3GC compared to the dry season. Conversely, a previous study carried out in North Africa characterized 3GC resistant Gram-negative bacteria in which a higher frequency of contamination of fruits and vegetables was identified during the rainy season (winter and fall for this region; Zekar et al., 2017). Other, previous research also found a higher load of microorganisms in vegetables during rainy seasons, due mainly to an increase in rainfall that carries garbage along with lower solar irradiation (Allende et al., 2017). The difference between our results could be due in Chile, the dry season is during the summer and spring, in which temperature of water could increase, river water flow could decrease, and rains are scarce, but if there is a rain event, this could drag accumulated material on the banks of the rivers, but this hypothesis should be further investigated. Moreover, none of the investigations mentioned before evaluated AMR. Very few studies have focused on AMR and environmental factors, one previous work has related the increase of bacteria (*E. coli* and *K. pneumoniae*) that carry ESBL in the human population during the summer, concluding that seasonality could play a fundamental role in the dissemination of ESBL (Wielders et al., 2020). These results agreed with those obtained in our work, as it is precisely in the summer that the highest number of ESBLs was found. While this study presented data for only 1year of sampling, longitudinal data for longer than 1year are necessary. Overall, understanding seasons with higher contamination with resistant Enterobacterales contribute to a better understanding of the transmission dynamics of AMR through food and to further develop interventions.

Our study indicates that performing screening on plates supplemented with ceftazidime increases the probability of finding MDR Enterobacterales in vegetable samples. Therefore, this antibiotic could be used as a marker for multidrug resistance in Enterobacterales in our region, agreeing with data from other regions, such as Europe, where cefotaxime is used as a marker of resistance (Shaw et al., 2021). Before this study, it was thought that supplementing with ciprofloxacin could have a similar effect on the detection of MDR Enterobacterales, as in this region a high prevalence of resistance to this antibiotic is reported (ISP, Instituto de Salud Publica, 2015; Durán, 2018). In addition, ciprofloxacin is usually found in aquatic environments, such as river water that is used to water vegetables due to its low biodegradation in aquatic environments (Girardi et al., 2011).

## Conclusion

This study emphasizes the importance of improving our understanding of environmental and food contamination with antimicrobial-resistant bacteria. The presence of MDR Enterobacterales isolates in vegetables that are mostly consumed without further cooking could represent a public health concern. MDR isolates, ESBL producers, and *mcr-1* were found in vegetables and river water that may irrigate those vegetables, these findings highlight the potential wide spread of MDR Enterobacterales and ESBL genes in the studied region. Because AMR is a global concern, reports from underrepresented regions in which environmental surveillance of AMR is not conducted could help to develop local awareness of AMR, especially for food-producing countries that may have underestimated the importance of environmental AMR.

## Data Availability Statement

The raw data supporting the conclusions of this article will be made available by the authors, without undue reservation.

## Author Contributions

CD-G, AM-S, AA, JO-P, and CB designed the study, conducted the experiments, and wrote the manuscript. AA ran the statistical analysis while CD-G constructed the heat map. LR, PG, and FA conducted experiments. GG-R, SC, AO-C, JM, and RA designed experiments and critically reviewed the manuscript.

## Funding

This work was funded by the FONDECYT 11160116 and 1181167, the ANID Millennium Science Initiative/ Millennium Initiative for Collaborative Research on Bacterial Resistance, MICROB-R, NCN17_081, and the FONDAP-ACCDIS grant number #15130011.

## Conflict of Interest

The authors declare that the research was conducted in the absence of any commercial or financial relationships that could be construed as a potential conflict of interest.

## Publisher’s Note

All claims expressed in this article are solely those of the authors and do not necessarily represent those of their affiliated organizations, or those of the publisher, the editors and the reviewers. Any product that may be evaluated in this article, or claim that may be made by its manufacturer, is not guaranteed or endorsed by the publisher.

## Footnotes

^1^ http://www.R-project.org

## References

[ref1] Allende A. Castro-Ibáñez I. Lindqvist R. Gil M. I. Uyttendaele M. Jacxsens L. (2017). Quantitative contamination assessment of *Escherichia coli* in baby spinach primary production in Spain: effects of weather conditions and agricultural practices. Int. J. Food Microbiol. 257, 238–246. doi: 10.1016/j.ijfoodmicro.2017.06.027, PMID: 28697385

[ref2] Benavides J. A. Salgado-Caxito M. Opazo-Capurro A. González Muñoz P. Piñeiro A. Otto Medina M.. (2021). ESBL-producing *Escherichia coli* carrying CTX-M genes circulating among livestock, dogs, and wild mammals in small-scale farms of Central Chile. Antibiotics 10:510. doi: 10.3390/antibiotics10050510, PMID: 33946277 PMC8145412

[ref3] Blaak H. van Hoek A. H. A. M. Veenman C. Docters van Leeuwen A. E. Lynch G. van Overbeek W. M.. (2014). Extended spectrum β-lactamase- and constitutively AmpC-producing *Enterobacteriaceae* on fresh produce and in the agricultural environment. Int. J. Food Microbiol. 168-169, 8–16. doi: 10.1016/j.ijfoodmicro.2013.10.006, PMID: 24211774

[ref4] Caltagirone M. Nucleo E. Spalla M. Zara F. Novazzi F. Marchetti V. M.. (2017). Occurrence of extended spectrum β-lactamases, KPC-type, and MCR-1.2-producing *Enterobacteriaceae* from wells, river water, and wastewater treatment plants in Oltrepò Pavese area, northern Italy. Front. Microbiol. 8:2232. doi: 10.3389/fmicb.2017.02232, PMID: 29176971 PMC5687051

[ref5] CDC, Centers for Disease Control and Prevention (2019). Antibiotic resistance threats in the United States. Available at: https://www.cdc.gov/drugresistance/pdf/threats-report/2019-ar-threats-report-508.pdf

[ref6] Cevidanes A. Esperón F. Cataldo S.Di Neves E. Sallaberry-Pincheira N. Millán J. (2020). Antimicrobial resistance genes in Andean foxes inhabiting anthropized landscapes in Central Chile. Sci. Total Environ. 724:138247. doi: 10.1016/j.scitotenv.2020.138247, PMID: 32268291

[ref7] CLSI, Clinical and Laboratory Standards Institute (2018). Performance Standards for Antimicrobial Susceptibility Testing. 28th *Edn*. Wayne, PA: Clinical and Laboratory Standards Institute.

[ref8] Dantas Palmeira J. Ferreira H. M. N. (2020). Extended-spectrum beta-lactamase (ESBL)-producing *Enterobacteriaceae* in cattle production – a threat around the world. Heliyon 6:e03206. doi: 10.1016/j.heliyon.2020.e03206, PMID: 32042963 PMC7002838

[ref9] De Oliveira D. M. P. Forde B. M. Kidd T. J. Harris P. N. A. Schembri M. A. Beatson S. A.. (2020). Antimicrobial resistance in ESKAPE pathogens. Clin. Microbiol. Rev. 33:e00181-19. doi: 10.1128/CMR.00181-19, PMID: 32404435 PMC7227449

[ref10] Diab M. Hamze M. Bonnet R. Saras E. Madec J. Y. Haenni M. (2018). Extended-spectrum beta-lactamase (ESBL)- and carbapenemase-producing *Enterobacteriaceae* in water sources in Lebanon. Vet. Microbiol. 217, 97–103. doi: 10.1016/j.vetmic.2018.03.007, PMID: 29615264

[ref11] Durán L. (2018). Resistencia antimicrobiana e implicancias para el manejo de infecciones del tracto urinario. Rev. Médica Clínica Las Condes 29, 213–221. doi: 10.1016/j.rmclc.2018.01.002

[ref12] Falomir M. P. Rico H. Gozalbo D. (2013). *Enterobacter* and *Klebsiella* species isolated from fresh vegetables marketed in Valencia (Spain) and their clinically relevant resistances to chemotherapeutic agents. Foodborne Pathog. Dis. 10, 1002–1007. doi: 10.1089/fpd.2013.1552, PMID: 23980710

[ref13] Ferronato N. Torretta V. (2019). Waste mismanagement in developing countries: a review of global issues. Int. J. Environ. Res. Public Health 16:1060. doi: 10.3390/ijerph16061060, PMID: 30909625 PMC6466021

[ref14] Girardi C. Greve J. Lamshöft M. Fetzer I. Miltner A. Schäffer A.. (2011). Biodegradation of ciprofloxacin in water and soil and its effects on the microbial communities. J. Hazard. Mater. 198, 22–30. doi: 10.1016/j.jhazmat.2011.10.004, PMID: 22036930

[ref15] Guo D. Thomas J. Lazaro A. Mahundo C. Lwetoijera D. Mrimi E.. (2019). Understanding the impacts of short-term climate variability on drinking water source quality: observations from three distinct climatic regions in Tanzania. GeoHealth 3, 84–103. doi: 10.1029/2018GH000180, PMID: 32159034 PMC7007091

[ref16] Guyomard-Rabenirina S. Dartron C. Falord M. Sadikalay S. Ducat C. Richard V.. (2017). Resistance to antimicrobial drugs in different surface waters and wastewaters of Guadeloupe. PLoS One 12:e0173155. doi: 10.1371/journal.pone.0173155, PMID: 28253356 PMC5333909

[ref17] Hernando-Amado S. Coque T. M. Baquero F. Martínez J. L. (2019). Defining and combating antibiotic resistance from one health and global health perspectives. Nat. Microbiol. 4, 1432–1442. doi: 10.1038/s41564-019-0503-9, PMID: 31439928

[ref18] Higuera-Llantén S. Vásquez-Ponce F. Barrientos-Espinoza B. Mardones F. O. Marshall S. H. Olivares-Pacheco J. (2018). Extended antibiotic treatment in salmon farms select multiresistant gut bacteria with a high prevalence of antibiotic resistance genes. PLoS One 13:e0203641. doi: 10.1371/journal.pone.0203641, PMID: 30204782 PMC6133359

[ref19] Hölzel C. S. Tetens J. L. Schwaiger K. (2018). Unraveling the role of vegetables in spreading antimicrobial-resistant bacteria: a need for quantitative risk assessment. Foodborne Pathog. Dis. 15, 671–688. doi: 10.1089/fpd.2018.2501, PMID: 30444697 PMC6247988

[ref20] Hudson J. A. Frewer L. J. Jones G. Brereton P. A. Whittingham M. J. Stewart G. (2017). The Agri-food chain and antimicrobial resistance: a review. Trends Food Sci. Technol. 69, 131–147. doi: 10.1016/j.tifs.2017.09.007

[ref21] ISP, Instituto de Salud Publica (2015). Boletin de Resistencia Antimicrobiana. 19. Available at: http://www.ispch.cl/sites/default/files/Boletin Gono-Final.pdf

[ref22] INE, Instituto Nacional de Estadísticas (2017). Resultado Censo 2017 para la región del Maule. Available at: http://resultados.censo2017.cl/Region?R=R07

[ref23] Iwu C. D. Okoh A. I. (2019). Preharvest transmission routes of fresh produce associated bacterial pathogens with outbreak potentials: a review. Int. J. Environ. Res. Public Health 16:4407. doi: 10.3390/ijerph16224407, PMID: 31717976 PMC6888529

[ref24] Jones R. N. Guzman-Blanco M. Gales A. C. Gallegos B. Castro A. L. L. Martino M. D. V.. (2013). Susceptibility rates in Latin American nations: report from a regional resistance surveillance program (2011). Braz. J. Infect. Dis. 17, 672–681. doi: 10.1016/j.bjid.2013.07.002, PMID: 24120834 PMC9427403

[ref25] Jongman M. Korsten L. (2016). Genetic diversity and antibiotic resistance of *Escherichia coli* isolates from different leafy green production systems. J. Food Prot. 79, 1846–1853. doi: 10.4315/0362-028X.JFP-16-117, PMID: 28221925

[ref26] Kolínská R. Španělová P. Dřevínek M. Hrabák J. Žemličková H. (2015). Species identification of strains belonging to genus *Citrobacter* using the biochemical method and MALDI-TOF mass spectrometry. Folia Microbiol. 60, 53–59. doi: 10.1007/s12223-014-0340-4, PMID: 25113540

[ref27] Lima T. Domingues S. Da Silva G. J. (2019). Plasmid-mediated colistin resistance in *Salmonella enterica*: a review. Microorganisms 7:55. doi: 10.3390/microorganisms7020055, PMID: 30791454 PMC6406434

[ref28] Liu G. Ali T. Gao J. Ur Rahman S. Yu D. Barkema H. W.. (2020). Co-occurrence of plasmid-mediated Colistin resistance (mcr-1) and extended-Spectrum ß-lactamase encoding genes in *Escherichia coli* from bovine Mastitic Milk in China. Microb. Drug Resist. 26, 685–696. doi: 10.1089/mdr.2019.0333, PMID: 31755810

[ref29] Liu C. Hofstra N. Franz E. (2013). Impacts of climate change on the microbial safety of pre-harvest leafy green vegetables as indicated by *Escherichia coli* O157 and Salmonella spp. Int. J. Food Microbiol. 163, 119–128. doi: 10.1016/j.ijfoodmicro.2013.02.026, PMID: 23558195

[ref30] Luna-Guevara J. J. Arenas-Hernandez M. M. P. Martínez De La Peña C. Silva J. L. Luna-Guevara M. L. (2019). The role of pathogenic *E. coli* in fresh vegetables: behavior, contamination factors, and preventive measures. Int. J. Microbiol. 2019:2894328. doi: 10.1155/2019/2894328, PMID: 31885595 PMC6899298

[ref31] Magiorakos A. P. Srinivasan A. Carey R. B. Carmeli Y. Falagas M. E. Giske C. G.. (2012). Multidrug-resistant, extensively drug-resistant and pandrug-resistant bacteria: an international expert proposal for interim standard definitions for acquired resistance. Clin. Microbiol. Infect. 18, 268–281. doi: 10.1111/j.1469-0691.2011.03570.x, PMID: 21793988

[ref32] Manage P. M. Liyanage G. Y. (2019). “Antibiotics induced antibacterial resistance,” in Pharmaceuticals and Personal Care Products: Waste Management and Treatment Technology (United Kingdom: Butterworth-Heinemann), 429–448.

[ref33] Manageiro V. Jones-dias D. Ferreira E. Caniça M. (2020). Plasmid-mediated colistin resistance (*mcr*-1) in *Escherichia coli* from non-imported fresh vegetables for human consumption in Portugal. Microorganisms 8:429. doi: 10.3390/microorganisms8030429, PMID: 32197505 PMC7143947

[ref34] Melo L. C. Oresco C. Leigue L. Netto H. M. Melville P. A. Benites N. R.. (2018). Prevalence and molecular features of ESBL/pAmpC-producing *Enterobacteriaceae* in healthy and diseased companion animals in Brazil. Vet. Microbiol. 221, 59–66. doi: 10.1016/j.vetmic.2018.05.017, PMID: 29981709

[ref35] Migura-Garcia L. González-López J. J. Martinez-Urtaza J. Aguirre Sánchez J. R. Moreno-Mingorance A. Perez de Rozas A.. (2020). *mcr*-colistin resistance genes mobilized by IncX4, IncHI2, and IncI2 plasmids in *Escherichia coli* of pigs and white stork in Spain. Front. Microbiol. 10:3072. doi: 10.3389/fmicb.2019.03072, PMID: 32010114 PMC6978640

[ref36] ODEPA, Oficina de Estudios y Políticas Agrarias (2018). Región del Maule, Infromación Regional 2018. Available at: https://www.odepa.gob.cl/wp-content/uploads/2018/06/Maule.pdf

[ref37] Oh S. S. Song J. Kim J. Shin J. (2020). Increasing prevalence of multidrug-resistant *mcr*-1-positive *Escherichia coli* isolates from fresh vegetables and healthy food animals in South Korea. Int. J. Infect. Dis. 92, 53–55. doi: 10.1016/j.ijid.2019.12.025, PMID: 31877351

[ref38] Pavez M. Troncoso C. Osses I. Salazar R. Illesca V. Reydet P.. (2019). High prevalence of CTX-M-1 group in ESBL-producing *Enterobacteriaceae* infection in intensive care units in southern Chile. Braz. J. Infect. Dis. 23, 102–110. doi: 10.1016/j.bjid.2019.03.002, PMID: 31028724 PMC9425662

[ref39] Rajabzadeh S. Bahreini M. Sharifmoghadam M. R. (2018). A rapid method for separating and concentration of food-borne pathogens using elution from ready-to-eat vegetables. Iran. J. Microbiol. 10, 385–393. PMID: 30873266 PMC6414744

[ref40] Rajendran B. Mutters N. T. Marasca G. Conti M. Sifakis F. Vuong C.. (2019). Mandatory surveillance and outbreaks reporting of the WHO priority pathogens for research & discovery of new antibiotics in European countries. Clin. Microbiol. Infect. 26, 943.e1–943.e6. doi: 10.1016/j.cmi.2019.11.020, PMID: 31812771

[ref41] Rebelo A. R. Bortolaia V. Kjeldgaard J. S. Pedersen S. K. Leekitcharoenphon P. Hansen I. M.. (2018). Multiplex PCR for detection of plasmid-mediated colistin resistance determinants, *mcr*-1, *mcr*-2, *mcr*-3, *mcr*-4 and *mcr*-5 for surveillance purposes. Euro Surveill. 23:17-00672. doi: 10.2807/1560-7917.ES.2018.23.6.17-00672, PMID: 29439754 PMC5824125

[ref42] Richter L. Du Plessis E. M. Duvenage S. Korsten L. (2019). Occurrence, identification, and antimicrobial resistance profiles of extended-spectrum and AmpC β-lactamase-producing *Enterobacteriaceae* from fresh vegetables retailed in Gauteng province, South Africa. Foodborne Pathog. Dis. 16, 421–427. doi: 10.1089/fpd.2018.2558, PMID: 30785775

[ref43] Sacristán I. Esperón F. Acuña F. Aguilar E. García S. López M. J.. (2020). Antibiotic resistance genes as landscape anthropization indicators: using a wild felid as sentinel in Chile. Sci. Total Environ. 703:134900. doi: 10.1016/j.scitotenv.2019.134900, PMID: 31757538

[ref44] Said B. L. Jouini A. Klibi N. Dziri R. Alonso C. A. Boudabous A.. (2015). Detection of extended-spectrum beta-lactamase (ESBL)-producing *Enterobacteriaceae* in vegetables, soil and water of the farm environment in Tunisia. Int. J. Food Microbiol. 203, 86–92. doi: 10.1016/j.ijfoodmicro.2015.02.023, PMID: 25791254

[ref45] Saksena R. Malik M. Gaind R. (2020). Bacterial contamination and prevalence of antimicrobial resistance phenotypes in raw fruits and vegetables sold in Delhi, India. J. Food Saf. 40, 1–8. doi: 10.1111/jfs.12739

[ref46] Salgado-Caxito M. Benavides J. A. Munita J. M. Rivas L. García P. Listoni F. J. P.. (2021). Risk factors associated with faecal carriage of extended-spectrum cephalosporin-resistant *Escherichia coli* among dogs in Southeast Brazil. Prev. Vet. Med. 190:105316. doi: 10.1016/j.prevetmed.2021.105316, PMID: 33725561

[ref47] Saliu E. M. Vahjen W. Zentek J. (2017). Types and prevalence of extended-spectrum beta-lactamase producing *Enterobacteriaceae* in poultry. Anim. Health Res. Rev. 18, 46–57. doi: 10.1017/S1466252317000020, PMID: 28641596

[ref48] Sapkota S. Adhikari S. Khadka S. Adhikari M. Kandel H. Pathak S.. (2019). Multi-drug resistant extended-spectrum beta-lactamase producing *Escherichia coli* and *Salmonella* on raw vegetable salads served at hotels and restaurants in Bharatpur, Nepal. BMC. Res. Notes 12:516. doi: 10.1186/s13104-019-4557-9, PMID: 31420003 PMC6697966

[ref49] Sbodio A. Maeda S. Lopez-Velasco G. Suslow T. V. (2013). Modified Moore swab optimization and validation in capturing *E. coli* O157: H7 and *Salmonella* enterica in large volume field samples of irrigation water. Food Res. Int. 51, 654–662. doi: 10.1016/j.foodres.2013.01.011

[ref50] Shaw L. P. Chau K. K. Kavanagh J. AbuOun M. Stubberfield E. Gweon H. S.. (2021). Niche and local geography shape the pangenome of wastewater-and livestock-associated *Enterobacteriaceae*. Sci. Adv. 7:eabe3868. doi: 10.1126/SCIADV.ABE3868, PMID: 33837077 PMC8034854

[ref51] Tanner W. D. VanDerslice J. A. Goel R. K. Leecaster M. K. Fisher M. A. Olstadt J.. (2019). Multi-state study of *Enterobacteriaceae* harboring extended-spectrum beta-lactamase and carbapenemase genes in U.S. drinking water. Sci. Rep. 9:3938. doi: 10.1038/s41598-019-40420-0, PMID: 30850706 PMC6408426

[ref52] Weller D. Belias A. Green H. Roof S. Wiedmann M. (2020). Landscape, water quality, and weather factors associated with an increased likelihood of foodborne pathogen contamination of New York streams used to source water for produce production. Front. Sustain. Food Syst. 3:124. doi: 10.3389/fsufs.2019.00124, PMID: 32440656 PMC7241490

[ref53] Wielders C. C. H. Van Duijkeren E. Van Den Bunt G. Meijs A. P. Dierikx C. M. Bonten M. J. M.. (2020). Seasonality in carriage of extended-spectrum β-lactamase-producing *Escherichia coli* and *Klebsiella pneumoniae* in the general population: a pooled analysis of nationwide cross-sectional studies. Epidemiol. Infect. 148:e68. doi: 10.1017/S0950268820000539, PMID: 32081112 PMC7118714

[ref54] WHO, World Health Organization (2019). Thirteenth general programme of work. Available at: www.iniscommunication.com

[ref55] Wu J. Yunus M. Islam M. S. Emch M. (2016). Influence of climate extremes and land use on fecal contamination of shallow tubewells in Bangladesh. Environ. Sci. Technol. 50, 2669–2676. doi: 10.1021/acs.est.5b05193, PMID: 26844955 PMC4775353

[ref56] Ye Q. Wu Q. Zhang S. Zhang J. Yang G. Wang J.. (2018). Characterization of extended-spectrum β-lactamase-producing *Enterobacteriaceae* from retail food in China. Front. Microbiol. 9:1709. doi: 10.3389/fmicb.2018.01709, PMID: 30135680 PMC6092486

[ref57] Yuan T. Vadde K. K. Tonkin J. D. Wang J. Lu J. Zhang Z.. (2019). Urbanization impacts the physicochemical characteristics and abundance of fecal markers and bacterial pathogens in surface water. Int. J. Environ. Res. Public Health 16:1739. doi: 10.3390/ijerph16101739, PMID: 31100947 PMC6572354

[ref58] Zekar F. M. Granier S. A. Marault M. Yaici L. Gassilloud B. Manceau C.. (2017). From farms to markets: gram-negative bacteria resistant to third-generation cephalosporins in fruits and vegetables in a region of North Africa. Front. Microbiol. 8:1569. doi: 10.3389/fmicb.2017.01569, PMID: 28883810 PMC5573783

[ref59] Zurfuh K. Poirel L. Nordmann P. Nüesch-Inderbinen M. Hächler H. Stephan R. (2016). Occurrence of the plasmid-borne *mcr*-1 colistin resistance gene in extended-spectrum-lactamase-producing *Enterobacteriaceae* in river water and imported vegetable samples in Switzerland. Antimicrob. Agents Chemother. 60, 2594–2595. doi: 10.1128/AAC.00066-16, PMID: 26883696 PMC4808203

