# Supplementary material for: Isolation of Ciprofloxacin and Ceftazidime-Resistant Enterobacterales From Vegetables and River Water Is Strongly Associated With the Season and the Sample Type
Source: Front Microbiol. 2021 Sep 14;12:604567. doi: 10.3389/fmicb.2021.604567 (PMC8477802; doi:10.3389/fmicb.2021.604567)
Supplement: Supplementary file 1 [file Data_Sheet_1.docx]

**Supplementary Figures and Tables**


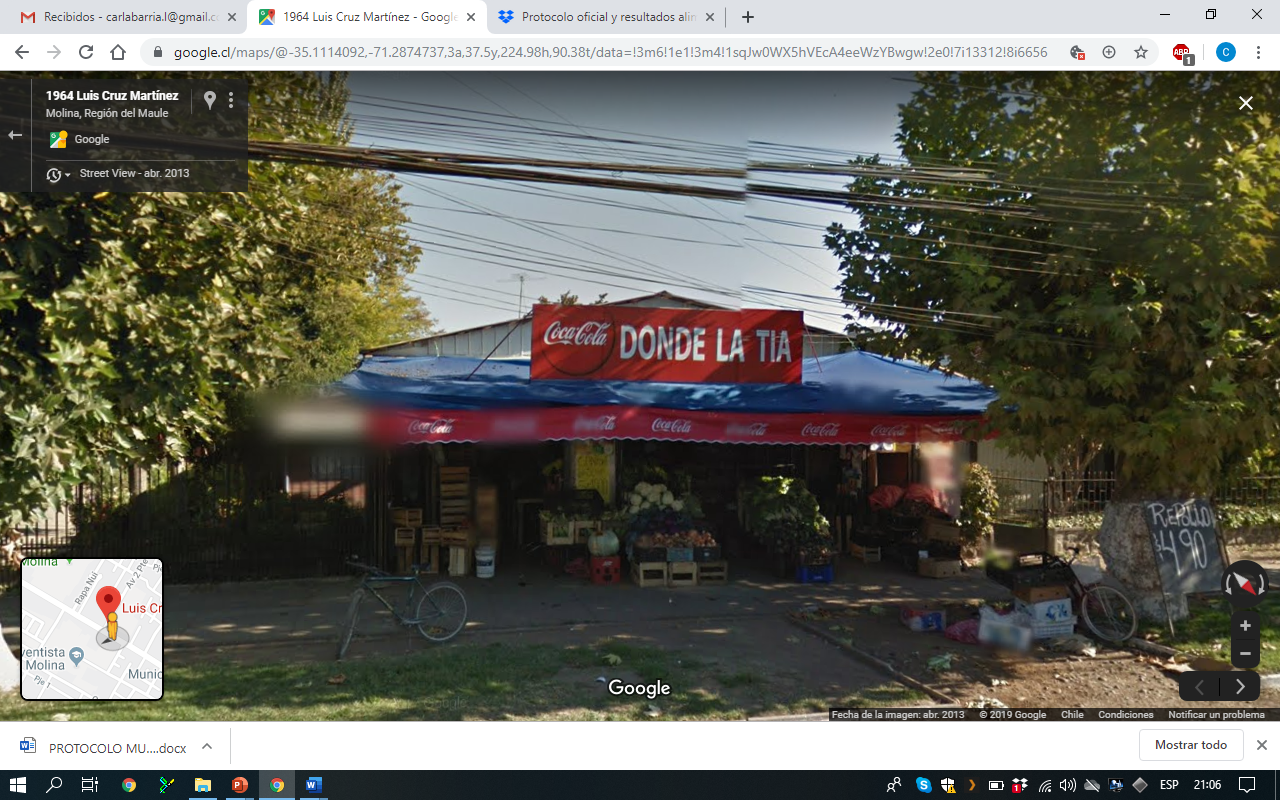

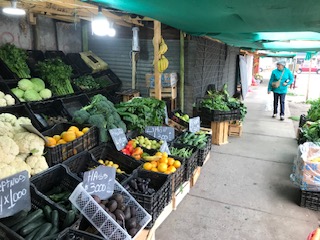

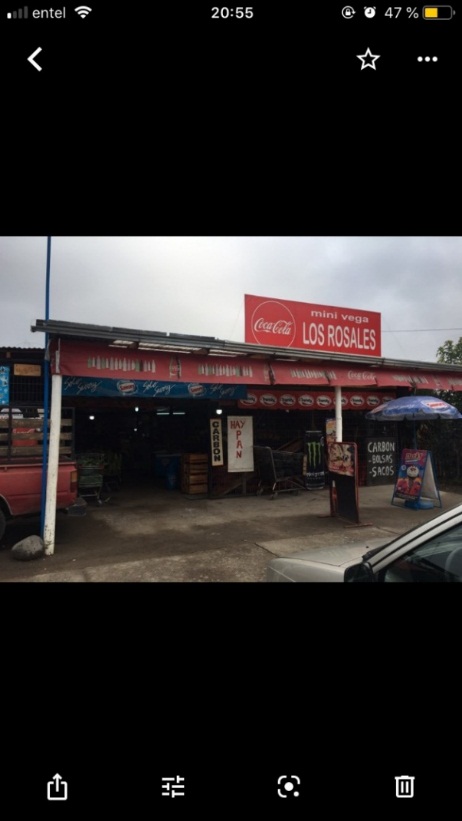


Figure S1**.** View of farmer markets tested in an agricultural town in central Chile.

Table S1. Antimicrobial susceptibility of 155 Enterobacterales isolated from vegetables and river collected during 2019-2020.

| Antimicrobial | Resistance (%) | Susceptibility (%) |
| --- | --- | --- |
| AMP^1^ | 74 (63.2) | 43 (36.8) |
| CFZ^2^ | 45 (38.5) | 72 (61.5) |
| CAZ | 54 (34.8) | 101 (65.2) |
| CRO | 53 (34.2) | 102 (65.8) |
| FEP | 25 (16.1) | 130 (83.9) |
| ETP | 21 (13.5) | 134 (86.5) |
| IPM | 3 (1.9) | 152 (98.1) |
| MEM | 0 (0) | 155 (100) |
| SAM^3^ | 31 (24.8) | 94 (75.2) |
| TZP | 13 (8.4) | 142 (91.6) |
| CIP | 115 (74.2) | 40 (25.8) |
| AMK | 3 (1.9) | 152 (98.1) |
| GEN | 5 (3.2) | 150 (96.8) |
| FOF | 4 (2.6) | 151 (97.4) |
| SXT | 65 (41.9) | 90 (58.1) |

^1^ For AMP 117 strains were tested. *C. freundii* and *K. sp* have intrinsic resistant.

^2^ For CFZ 117 strains were tested. *C. freundii*, *K. aerogenes* and *E. cloacae* have intrinsic resistant.

^3^ For SAM 125 strains were tested. *C. freundii* and *K. aerogenes* have intrinsic resistant.


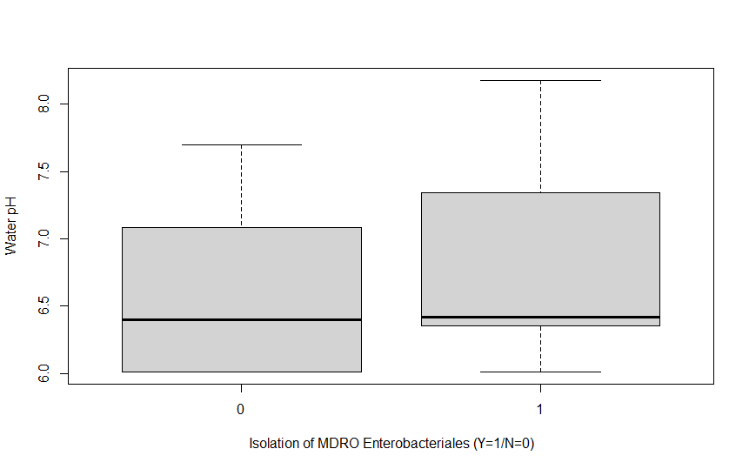

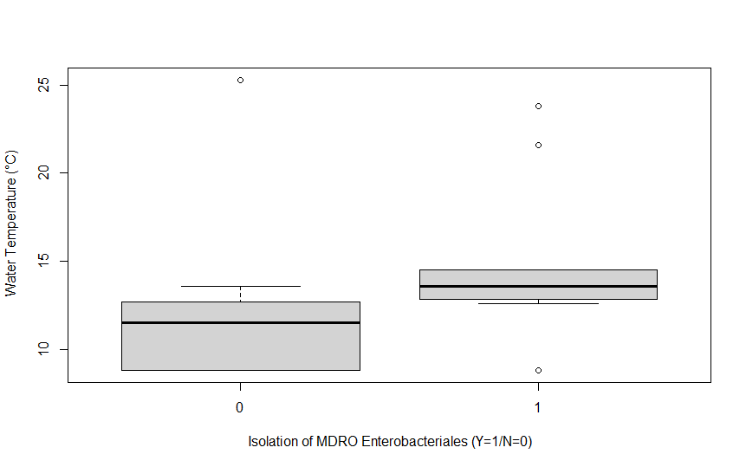

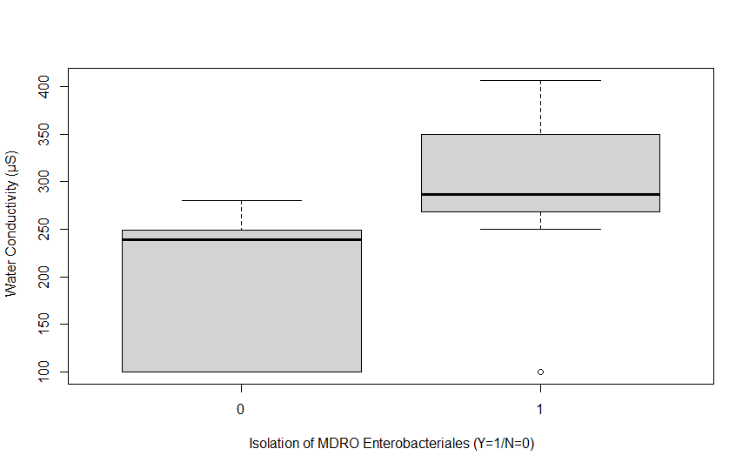

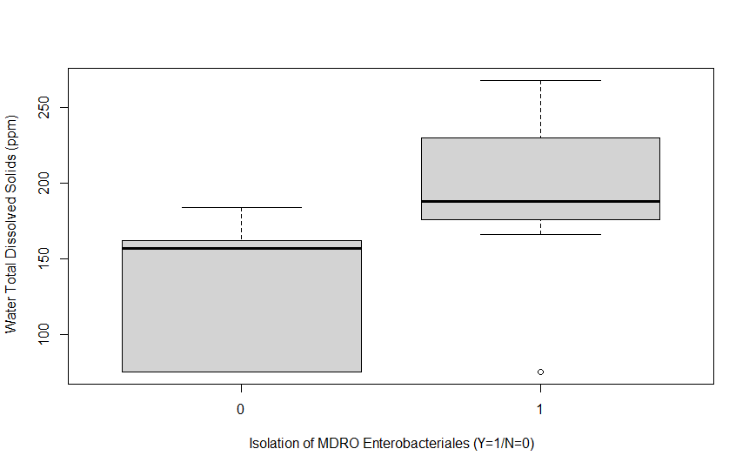

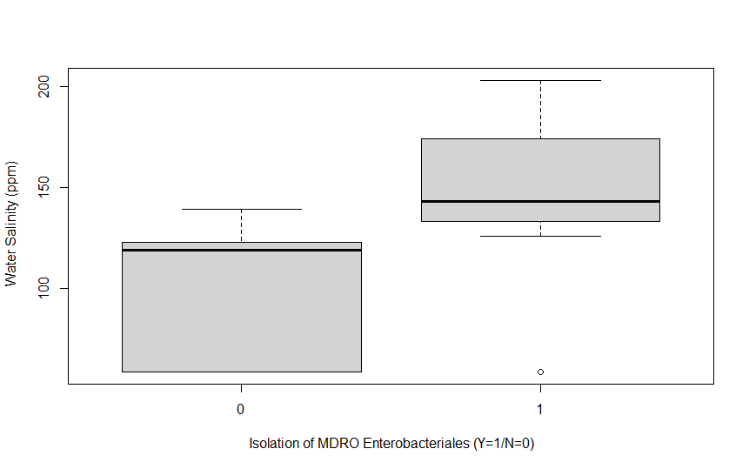

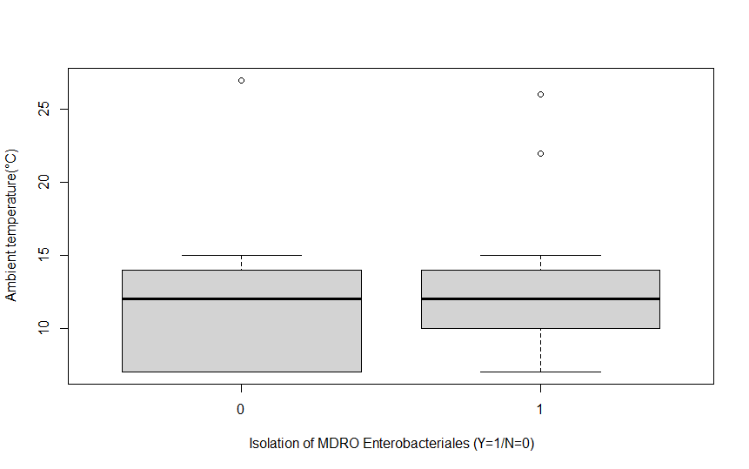


A

B

C

D

E

F

Figure S2. Descriptive statistics of the isolation of MDR Enterobacterales in river water samples based on continuous environmental factors recorded when the sample was been collected, where represents isolates detected based on (A) water pH, (B) water temperature (°C), (C) water conductivity (µS), (D) water total dissolved solids (TDS, ppm), (E) water salinity (ppm), and (F) ambient temperature (°C)


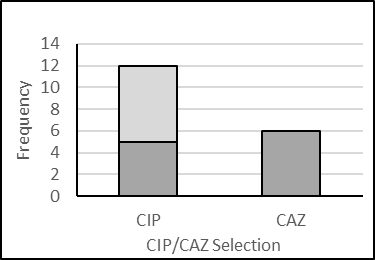

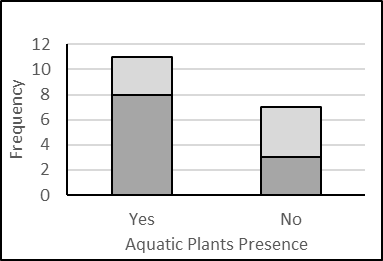

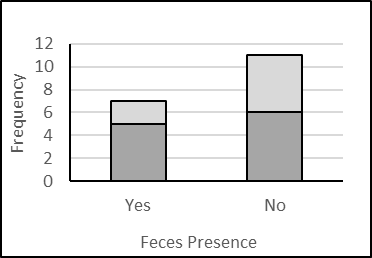

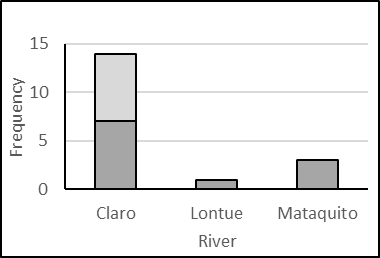

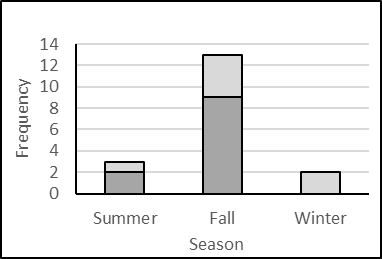


B


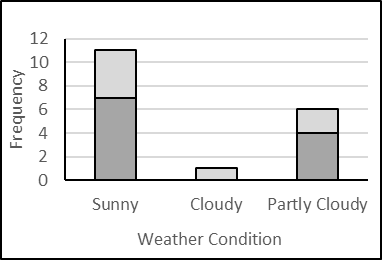

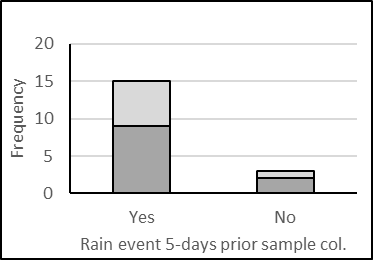

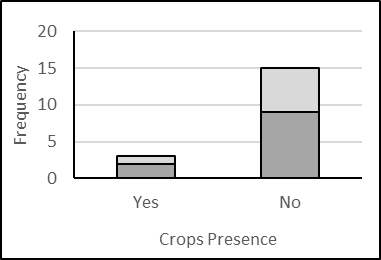

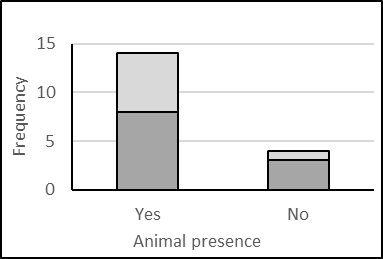

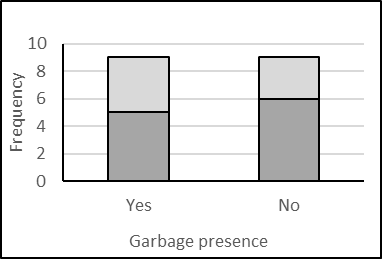


G

H

I

F

E

D

A

J

C

Figure S3. Descriptive statistics of the isolation of MDR Enterobacterales in river water samples based on categorical environmental factors recorded when the sample was been collected, where represents isolates detected based on (A) river (Lontue, Mataquito, and Claro rivers), (B) season (Fall, Summer, and Spring, no MDRO isolates were detected in winter), (C) antibiotic used to select *Enterobacteriaceae isolates* (CIP or CAZ), (D) Weather condition (sunny, partly cloudy, or cloudy, there was no rainy days during sample collection), (E) sight of crops nearby the sampling site (Yes/No), (F) rain event 5-days prior the sample collection (Yes/No), (G) animal presence when collecting the sample (Yes/No), (H) garbage presence when collecting samples (Yes/No), (I) feces presence when collecting samples, and (J) aquatic plants presence in sampling point. Dark grey bars represent isolation of MDR *Enterobacteriales*, while light grey bars indicate that there was no isolation of MDR *Enterobacteriales*.


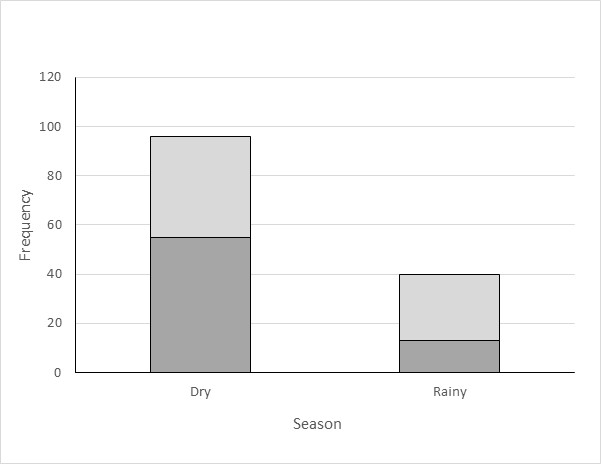


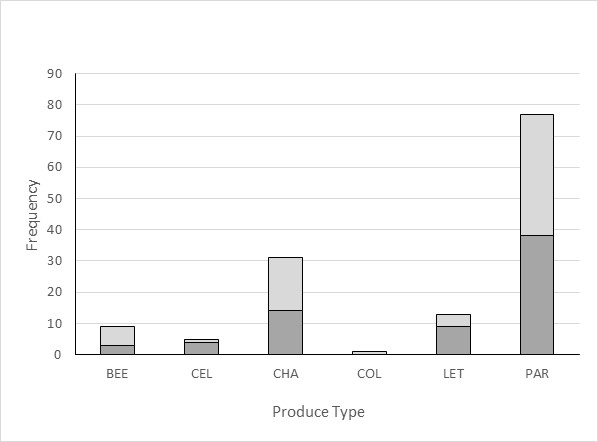

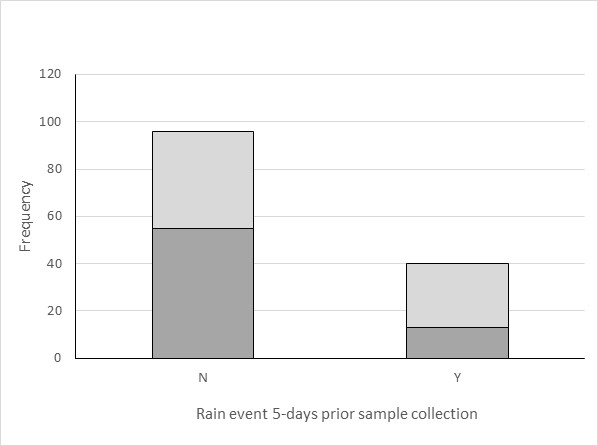

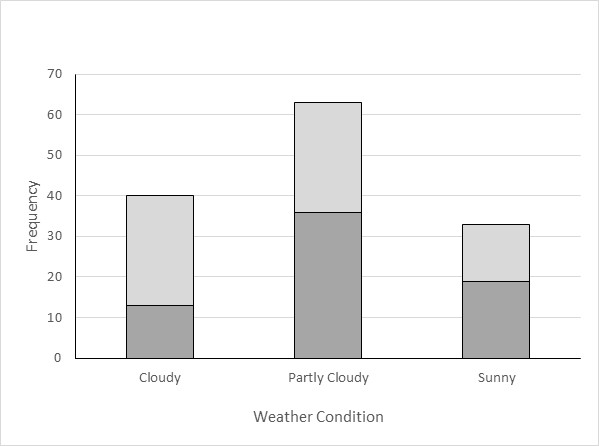

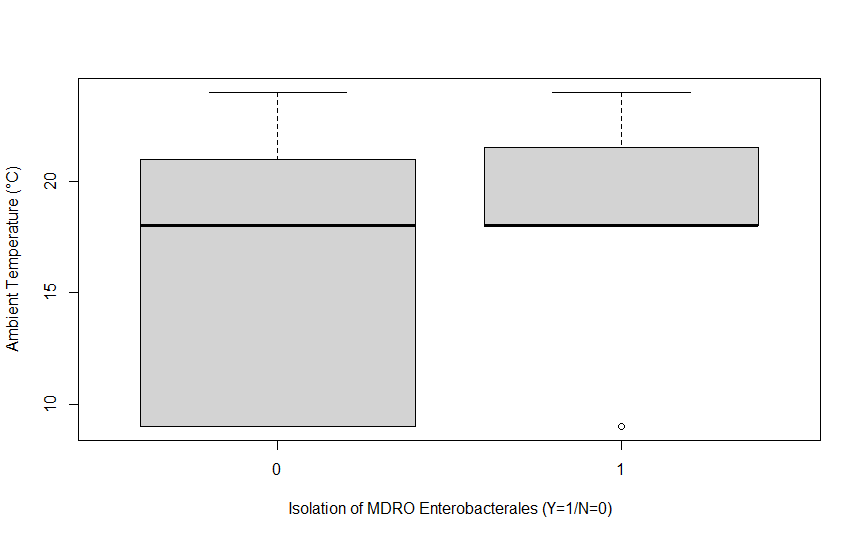

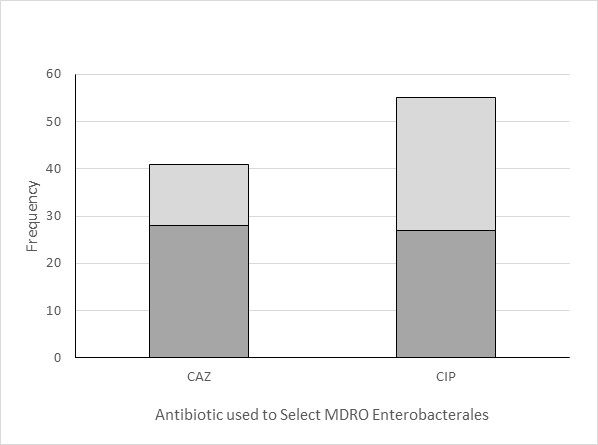


A

B

C

D

E

F

Figure S4: Descriptive statistics of the isolation of MDR *Enterobacterales* in vegetable samples collected from May 2019 to January 2020 based on categorical (bar plot) or continuous (box plot) environmental factors recorded when the sample was been collected, where represents isolates detected based on (A) type of produce (Beet, Celery, Chard, Coriander, lettuce, and Parsley), (B) season (dry or rainy), (C) rain event 5-days prior the sample collection (Yes/No), (D) Weather condition (sunny, partly cloudy, or cloudy, there was no rainy days during sample collection), and (E) antibiotic used to select *Enterobacteriaceae isolates* (CIP or CAZ), and (E) sight of crops nearby the sampling site (Yes/No), (F) ambient temperature (°C). In the bar plots, dark grey bars represent isolation of MDR *Enterobacteriales*, while light grey bars indicate that there was no isolation of MDR *Enterobacteriales*.
